# Piezo1 specific deletion in macrophage protects the progression of liver fibrosis in mice

**DOI:** 10.7150/thno.86103

**Published:** 2023-10-02

**Authors:** Shangfei Luo, Xiaoduo Zhao, Jintao Jiang, Bo Deng, Silin Liu, Honglin Xu, Qiaorui Tan, Yu'an Chen, Ziyan Zhang, Xianmei Pan, Rentao Wan, Xiaoting Chen, Youfen Yao, Jing Li

**Affiliations:** 1 Lingnan Medical Research Center, Guangzhou University of Chinese Medicine, Guangzhou, 510405, China.; 2 The First Affiliated Hospital, Guangzhou University of Chinese Medicine, Guangzhou, 510405, China.; 3 Department of Pathology, the First Affiliated Hospital of Zhejiang University School of Medicine, Hangzhou, 310006, China.; 4 The Second Affiliated Hospital of Guangzhou Medical University, Guangzhou Medical University, Guangzhou, 510260, China.; 5 Innovation Research Center, Shandong University of Chinese Medicine, Jinan, 250307, China.; 6 School of Biomedical Sciences, Faculty of Biological Sciences, University of Leeds, LS2 9JT, UK.

**Keywords:** liver fibrosis, Piezo1, macrophage, inflammation, cathepsin S

## Abstract

**Background and Aims:** Liver fibrosis is the common pathological pathway of chronic liver diseases and its mechanisms of which have not been fully declared. Macrophages play essential roles in progression of liver fibrosis partially by sensing abnormal mechanical signals. The aim of the study is to investigate the functions of macrophage Piezo1, a mechano-sensitive ion channel, in liver fibrosis.

**Approach and Results:** Immunofluorescence in human and murine fibrotic liver samples revealed that expression of macrophage Piezo1 was increased. Myeloid-specific *Piezo1* knockout (*Piezo1^ΔLysM^*) attenuated liver fibrosis by decreased collagen deposition and epithelial-mesenchymal transition (EMT). In *Piezo1^ΔLysM^* mice, less inflammation during development of liver fibrosis was observed by lessened macrophage infiltration, decreased M1 polarization and expression of inflammatory cytokines. RNA-seq data showed macrophage Piezo1 regulated transcription of cathepsin S (CTSS). *Piezo1^ΔLysM^* inhibited expression and activity of CTSS *in vitro* and* in vivo* and regulated T cell activity. Furthermore, inhibition of CTSS reversed macrophage inflammatory response driven by Piezo1 activation and LPS. Macrophage Piezo1 activation promoted CTSS secretion due to increased activity of Ca^2+^-dependent calpain protease induced by Ca^2+^ influx to cleave lysosome-associated membrane protein-1 (LAMP1). Pharmacological inhibition of calpain activity partially blocked Piezo1 mediated CTSS secretion.

**Conclusions:** Macrophage Piezo1 deficiency limits the progression of liver fibrosis by inhibited inflammatory response and decreased secretion of CTSS. These findings suggest that targeting Piezo1 channel may be a potential strategy for treating hepatic fibrosis.

## Introduction

Chronic liver inflammation caused by various factors such as cholestasis, virus infection, alcohol, non-alcoholic steatohepatitis or autoimmune diseases leads to liver fibrosis 1, 2. Liver fibrosis is a complex process that can either progress to cirrhosis and hepatocellular carcinoma (HCC) or potentially be reversed 3. Abnormal repetitive wound healing and uncontrolled inflammation are regarded as the characteristics of liver fibrosis, leading to excessive collagen deposition produced by activated myofibroblasts in extracellular matrix (ECM) 1. Immune cells, especially macrophages, including monocyte-derived macrophages and Kupffer cells, play an essential role in the process of hepatic fibrosis 4, 5. The infiltrating macrophages activate to proinflammatory phenotype and release cytokines to damage hepatocytes and activate hepatic stellate cells during liver fibrosis, while Ly-6C^low^ macrophages show restorative phenotype by secreting matrix metalloproteinases (MMPs)^6^-^8^. Thus, understanding the underlying mechanisms of macrophage in liver fibrosis are necessary for targeted therapy in ongoing liver fibrosis.

Under physiological conditions, cells sense multiple mechanical stimuli, including shear stress, compression, etc., which play an essential role in maintaining their functions. Pathological mechanical signals, such as increased tissue stiffness and portal hypertension were observed in the progression of hepatic fibrosis 1, 9, 10. Normal stiffness of human liver is about 5 kPa, while the liver stiffness could exceed 30 kPa in human cirrhotic livers 11, 12. Piezo1 was first discovered in 2010 13. As a mechanically sensitive cation channel protein, activation of Piezo1 allows influx of K^+^, Na^+^, Ca^2+^, and Mg^2+^ (with a certain preference for Ca^2+^) 14. The important roles of Piezo1 were identified in pathophysiological processes of fibrosis, including renal fibrosis 15, 16, cardiac fibrosis 17, 18, pulmonary fibrosis 19, 20 and others 21-23. Macrophages regulate their biological functions by sensing micro-environments of varied stiffness 24, 25. Compared with other mechanosensory ion channels, high expression of Piezo1 was detected in macrophages 19, 26. Macrophage Piezo1 could regulate iron metabolism through modulating phagocytic activity 27. Additionally, a recent study unveiled liver sinusoidal endothelial cells could sense cyclic stretch via Piezo1, subsequently facilitating neutrophil recruitment and promoting portal hypertension 28. However, how macrophage Piezo1 influences liver fibrosis has not been reported.

Cathepsin S (CTSS), a lysosomal protease, is mainly expressed in macrophages and dendritic cells 29. Its functions are best known for antigen processing and ECM remodeling 30, 31. Inhibition of CTSS could attenuate inflammatory response in macrophages during liver injury 32. Furthermore, the secretion of CTSS can be controlled by intracellular Ca^2+^ concentration and inhibition of extracellular CTSS activity could reverse intestinal fibrosis 33, 34.

In this study, we aimed to investigate the functions of macrophage Piezo1 in hepatic fibrosis. First, expression of Piezo1 was measured in fibrotic liver samples of human and mice. Second, the expression of macrophage Piezo1 was detected in human and murine fibrotic liver samples and myeloid-specific deletion of *Piezo1* (*Piezo1^ΔLysM^*) mice were generated to elucidate functions of macrophage Piezo1 in liver fibrosis induced by bile duct ligation (BDL) and carbon tetrachloride (CCl_4_) injection. Third, we explored the mechanisms of macrophage Piezo1 in regulating hepatic fibrosis and inflammation.

## Methods

### Human liver samples

Liver biopsy samples from patients who underwent necessary pathological diagnosis were obtained by hepatectomy. The diagnosis of liver fibrosis was confirmed by Hematoxylin & Eosin (H&E) and Masson's staining. A total of 20 cases were collected, including 3 patients suffered from colorectal adenocarcinoma with liver metastasis (liver function test was normal and postoperative pathological section revealed no obvious liver fibrosis in adjacent tissues of the metastatic tumor), 4 patients with hepatitis B cirrhosis-related hepatocellular carcinoma (HCC), 5 patients with primary biliary cirrhosis (PBC), 4 patients with biliary atresia (BA) and 4 patients with cholangiectasis and hepatolithiasis. Control samples were acquired from the edge of metastasis tumor in liver tissues. The detailed information of patients was shown in Table S1.

### Animals

About 6-8-week-old male mice were maintained under a SPF environment at appropriate temperature and humidity on 12 h light/12 h dark cycle with normal diet. BDL and CCl_4_ injection were used to induce murine liver fibrosis. For BDL, mice were anesthetized by 2% isoflurane inhalation and the abdominal incision was opened through midline. The common bile duct was separated, then ligated with 5-0 operative suture at two different locations. The mice were sacrificed 14 days after the surgery. For CCl_4_-induced liver fibrosis, mice were intraperitoneal injected olive oil or 2 mL/kg CCl_4_ dissolved in olive oil (1:4 volume ratio) three times a week for 4 weeks. The mice were killed 24 h after last injection.

### Data analysis

All data were presented as mean ± S.E.M. Tests for statistical significance were performed using the SPSS 20 software and the graphs were managed by OriginPro 2018. Student's *t-test* or Mann-Whitney U test were used to compare the two data sets. One-way ANOVA followed by Bonferroni multiple comparison tests were performed in some data sets. *P*-value < 0.05 was considered statistically significant.

Detailed methods are described in the supplemental materials.

## Results

### Increasing macrophage Piezo1 expression is correlated with liver fibrosis

**Figure 1:**
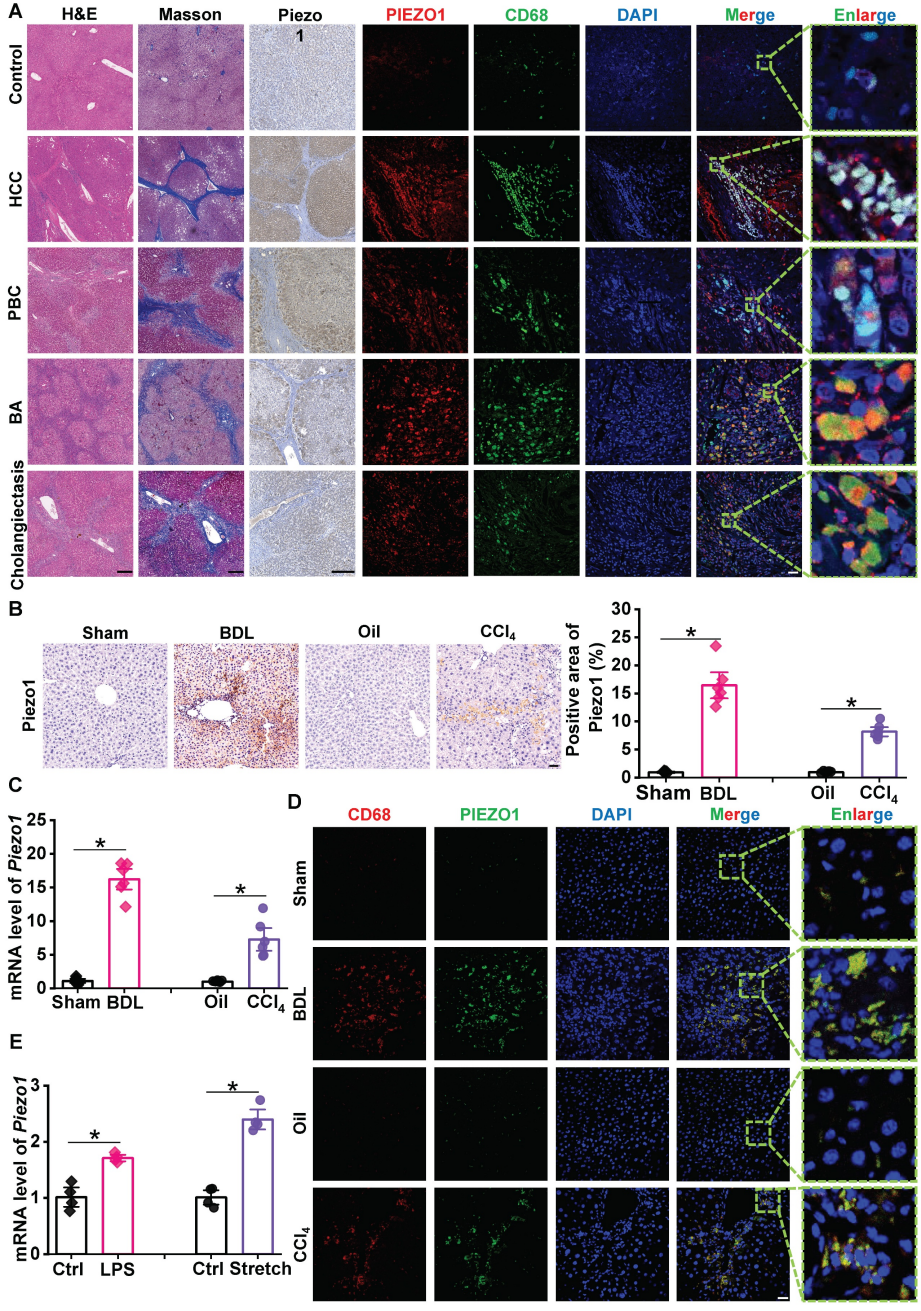
** Expression of Piezo1 in macrophage has increased in fibrotic livers. (A)** Representative images of H&E, Masson's, immunochemistry staining of Piezo1 and dual immunofluorescence staining with CD68 (green) and Piezo1 (red) in human liver samples. Scale bar, H&E and Masson's, 400 μm; immunochemistry, 200 μm; immunofluorescence, 50 μm, enlarge, 5.75 μm. **(B)** Representative images of Piezo1 staining and quantification of positive area in liver sections of C57BL/6J mice. Scale bar, 50 μm. **(C)** Relative mRNA expression of *Piezo1* in liver tissues of C57BL/6J mice. **(D)** Representative images of dual immunofluorescence staining with CD68 (red) and Piezo1 (green) in liver sections of C57BL/6J mice. Scale bar, 25 μm; enlarge, 5 μm. **(E)** Relative mRNA expression of *Piezo1* in BMDMs isolated from C57BL/6J mice. Data are presented as mean ± S. E. M.; Human samples: control (n = 3), HBV-related HCC (n = 4), PBC (n = 5), BA (n = 4), cholangiectasis (n = 4); mice samples (n = 6 for each group); cell samples (n = 4 for each group). *P < 0.05.

To investigate Piezo1 expression in liver fibrosis, we collected 20 human liver samples for slice staining. H&E and Masson's staining were used to confirmed the diagnosis of liver fibrosis in human liver samples. Compared with control samples, immunochemistry staining showed that Piezo1 expression was remarkedly elevated in human fibrotic liver samples (Figure 1A, left part). Then C57BL/6J mice subjected to BDL surgery and CCl_4_ injection, immunochemistry staining and RT-qPCR showed Piezo1 expression was markedly increased in BDL and CCl_4_ model (Figure 1B-C).

To determine participation of Piezo1 in liver fibrosis, *Piezo1^+/+^* (wildtype) and *Piezo1^+/-^* (heterozygous) mice were used to induce liver fibrosis. The progression of liver fibrosis, which was detected by H&E, Masson's and Sirius red staining, were significantly slowed down in *Piezo1^+/-^* mice compared with that in *Piezo1^+/+^* mice (Figure S2A-C). Similarly, serum alanine aminotransferase (ALT) and aspartate aminotransferase (AST) in hepatic fibrotic *Piezo1^+/-^* mice were significantly lower than *Piezo1^+/+^* mice (Figure S2D-E). These data showed that Piezo1 may involve in liver fibrosis.

Next, we determined whether Piezo1 expression increased in liver macrophages. Immunofluorescence of Piezo1 and CD68 in liver sections of human samples showed a significant accumulation of CD68^+^ cells in human fibrotic livers, while the labeling of Piezo1 in these cells were significantly stronger than control samples. (Figure 1A, right part). The specificity of the Piezo1 antibody was measured in slices of human fibrotic liver samples (Figure S1). Also, consistent with human fibrotic livers, the number of CD68^+^ cells and their intensity of Piezo1 in liver sections were markedly increased in BDL-subjected and CCl_4_-injected C57BL/6J mice (Figure 1D). In addition, bone marrow-derived macrophages (BMDMs) isolated from C57BL/6J mice were treated with physical stimuli (mechanical stretch) or chemical stimuli (LPS). The mRNA expression of *Piezo1* in stretch or LPS-treated BMDMs were significantly elevated compared with their controls (Figure 1E). These finding indicated that macrophage Piezo1 may play an essential role in liver fibrosis.

### Myeloid Piezo1 deletion attenuates liver fibrosis

**Figure 2:**
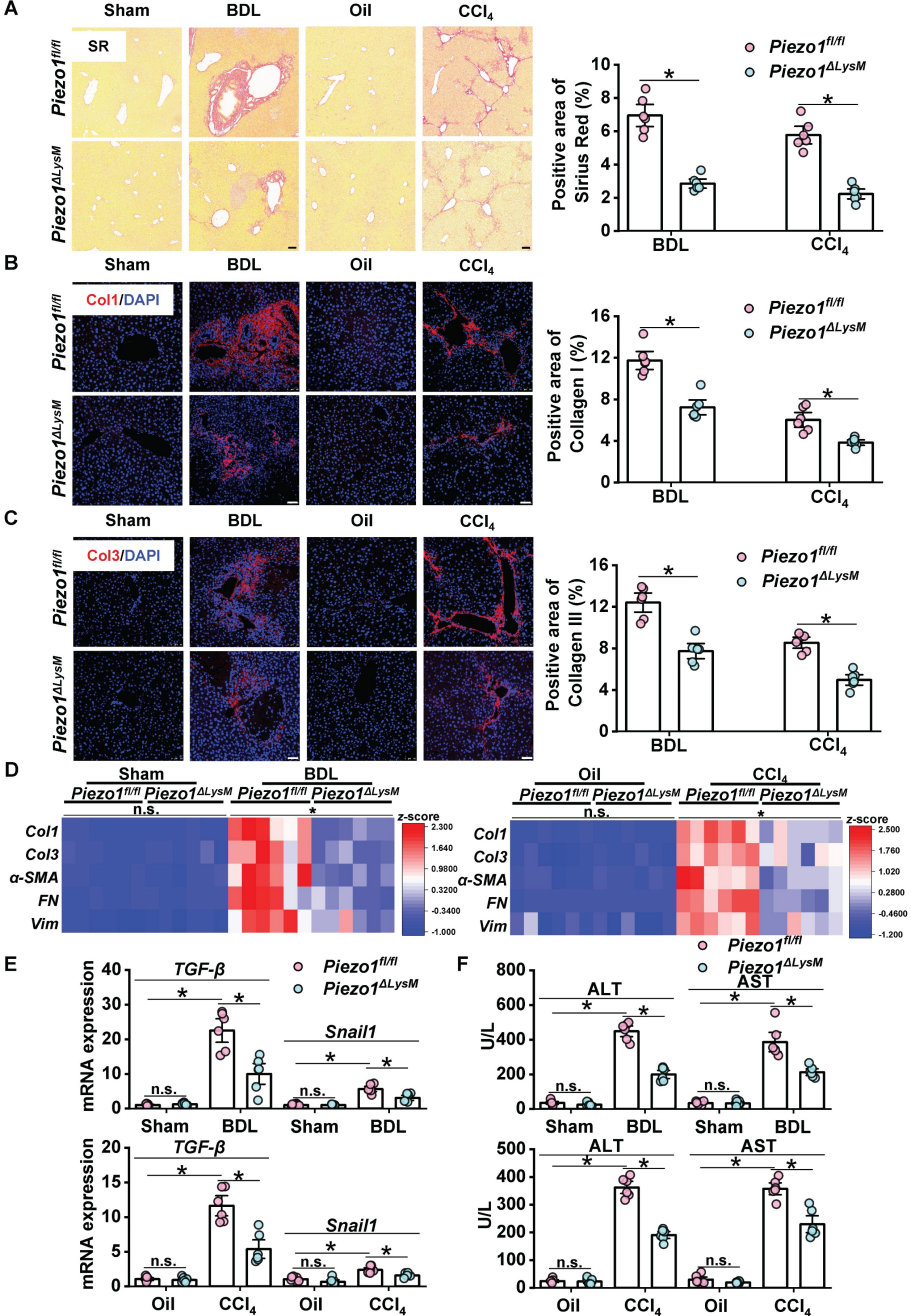
** Myeloid *Piezo1* deletion attenuates liver fibrosis. (A)** Representative images of Sirius red staining in liver sections of *Piezo1^fl/fl^* and *Piezo1^ΔLysM^* mice. Scale bar, 100 μm. Immunofluorescence staining of** (B)** collagen I (Col1, red) and **(C)** collagen III (Col3, red) in liver sections of *Piezo1^fl/fl^* and *Piezo1^ΔLysM^* mice were detected. Scale bar, 50 μm. **(D)** Relative mRNA levels of *Col1*, *Col3*, *α-SMA*, *FN* and *Vim* in liver tissues of *Piezo1^fl/fl^* and *Piezo1^ΔLysM^* mice were measured. Heatmap shown the z-score of gene levels. **(E)** Relative mRNA levels of *TGF*-*β* and *Snail1* in liver tissues of *Piezo1^fl/fl^* and *Piezo1^ΔLysM^* mice were quantified. **(F)** Serum concentrations of ALT and AST were measured. Data are presented as mean ± S. E. M.; n = 6 for each group; *P < 0.05.

In order to investigate functions of macrophage Piezo1 in liver fibrosis, myeloid-specific *Piezo1* deficiency mice (*Piezo1^ΔLysM^*) were used in this study. *Piezo1^fl/fl^* and *Piezo1^ΔLysM^* BMDMs were used to validate Piezo1 deletion in myeloid cells (Figure S3A-D). To examine whether myeloid Piezo1 knockout influence the process of liver fibrosis, *Piezo1^fl/fl^* and *Piezo1^ΔLysM^* littermates were treated with BDL surgery or CCl_4_ injection. The mRNA expression of *Piezo1* in fibrotic livers of *Piezo1^ΔLysM^* mice were extremely decreased compared with BDL-operated and CCl_4_-injected *Piezo1^fl/fl^* mice (Figure S4A). Sirius red and immunofluorescence staining of collagen I (Col1) and collagen III (Col3) which showed the formation of fibrosis in liver sections of *Piezo1^ΔLysM^* mice were extremely decreased compared with BDL-operated and CCl_4_-injected *Piezo1^fl/fl^* mice (Figure 2A-C). Also, immunochemistry staining of alpha-smooth muscle actin (α-SMA), fibronectin and vimentin, which are regarded as EMT markers, showed lower expression in fibrotic livers of *Piezo1^ΔLysM^* mice than those *Piezo1^fl/fl^* littermates (Figure S5A-C). Similarly, lower mRNA levels of *Col1*, *Col3*, *α-SMA*, fibronectin (*FN*) and vimentin (*Vim*), transforming growth factor (*TGF*)-β and *Snail1* in fibrotic livers of *Piezo1^ΔLysM^* mice than *Piezo1^fl/fl^* littermates were observed (Figure 2D-E). In addition, higher serum ALT and AST were measured in hepatic fibrotic *Piezo1^fl/fl^* mice than *Piezo1^ΔLysM^* littermates (Figure 2F). Taking together, our results showed Piezo1 knockout in myeloid cells attenuate liver fibrosis.

### Myeloid-specific *Piezo1* regulates macrophage infiltration in murine fibrotic livers

**Figure 3:**
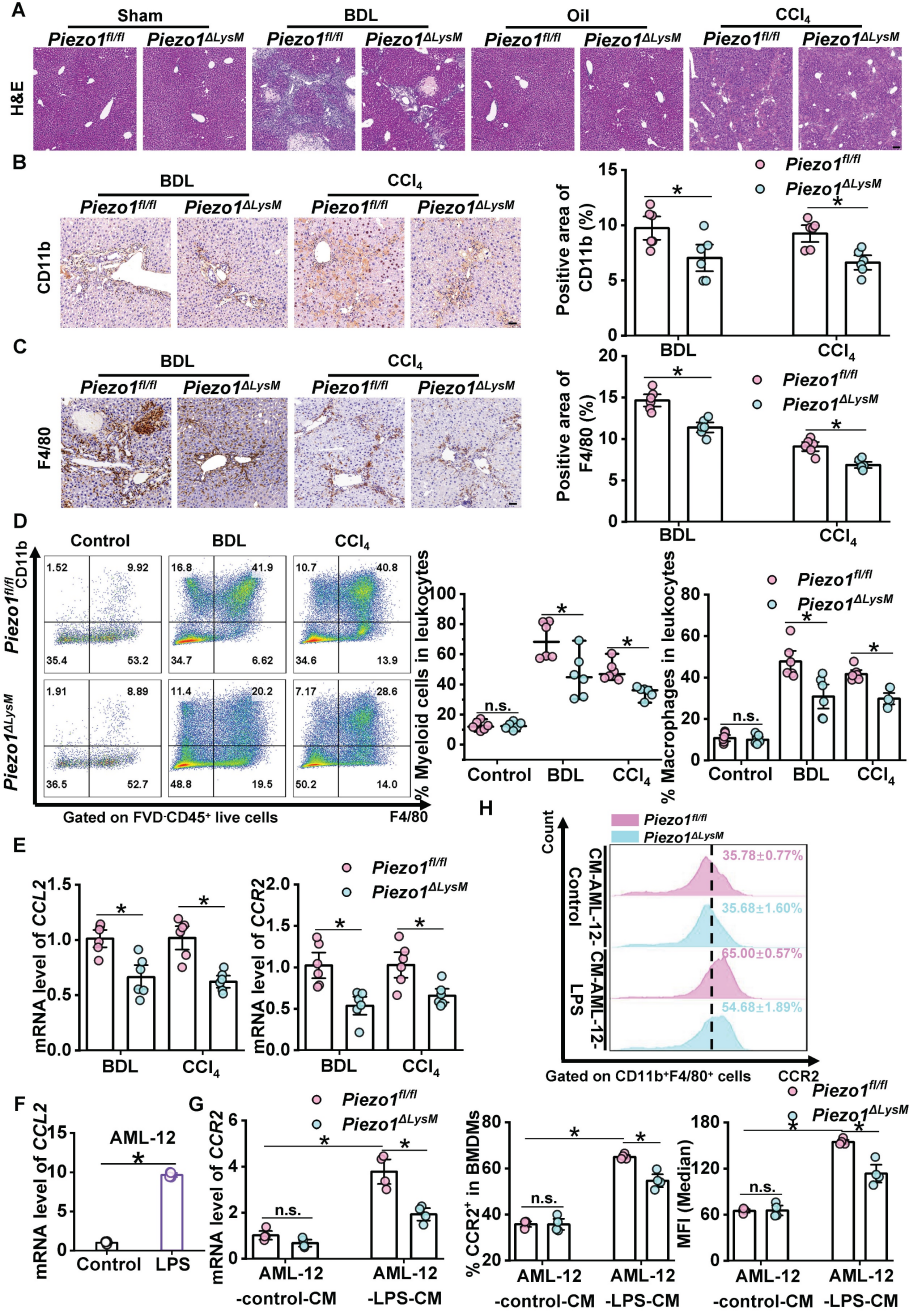
** Myeloid-specific *Piezo1* regulates macrophage infiltration in murine fibrotic livers. (A)** Representative images of H&E in liver sections of *Piezo1^fl/fl^* and *Piezo1^ΔLysM^* mice. Scale bar, 100 μm. Immunohistochemistry staining of** (B)** CD11b and **(C)** F4/80 were detected in liver sections of *Piezo1^fl/fl^* and *Piezo1^ΔLysM^* mice. Scale bar, 50 μm. **(D)** Total live myeloid cells (FVD*^-^*CD45*^+^*CD11b*^+^*) and macrophages (FVD*^-^*CD45*^+^*CD11b*^+^*F4/80*^+^*) were quantified in livers of *Piezo1^fl/fl^* and *Piezo1^ΔLysM^* mice by flow cytometry (FCM). **(E)** Relative mRNA expression levels of *CCL2* and *CCR2* in liver tissues of *Piezo1^fl/fl^* and *Piezo1^ΔLysM^* mice were measured. **(F)** Relative mRNA expression of *CCL2* in AML-12 were measured. **(G)** Relative mRNA expression of *CCR2* in BMDMs were measured.** (H)** CCR2^+^ cells in BMDMs and its median fluorescence intensity (MFI) were quantified by FCM. Data are presented as mean ± S. E. M.; mice samples: n = 6 for each group; cell samples: n = 4 for each group; *P < 0.05.

We examined whether myeloid-specific *Piezo1* effects on macrophage infiltration during liver fibrosis. H&E staining showed infiltration of immune cells in hepatic fibrotic *Piezo1^ΔLysM^* mice were less than *Piezo1^fl/fl^* littermates (Figure 3A). Immunochemistry staining of CD11b and F4/80 showed significant accumulation of positive cells in fibrotic livers of *Piezo1^fl/fl^* mice compared with* Piezo1^ΔLysM^* littermates (Figure 3B-C). Then, we assessed the population of myeloid cells isolated from murine livers and spleens using flow cytometry (FCM). Figure S6A and Figure S7A showed the gating strategy of hepatic and splenic myeloid cells, including neutrophils and macrophages. Compared with fibrotic livers of *Piezo1^fl/fl^* mice, the frequency of hepatic myeloid cells and macrophages were markedly reduced in fibrotic livers of *Piezo1^ΔLysM^* littermates (Figure 3D). However, no significant differences were observed in the abundance of neutrophils between the two groups (Figure S6B). No disparity in the percentage of splenic myeloid cells, macrophages and neutrophils were detected between the experimental groups (Figure S7B)**.**

Monocyte chemoattractant protein-1 (CCL2) can mediate C-C chemokine receptor-2 (CCR2)^+^ macrophage infiltration to promote liver fibrosis 35. We found that the mRNA levels of *CCL2* and *CCR2* were substantially increased in fibrotic livers of *Piezo1^fl/fl^* mice compared with *Piezo1^ΔLysM^* littermates (Figure 3E). A recent study revealed that injured hepatocytes expressed CCL2 36. We treated AML-12 cells with LPS to induce injured state. The expression of *CCL2*, as assessed by RT-qPCR, was significantly elevated in LPS-treated AML-12 cells compared with the control group (Figure 3F). Next, the conditioned culture medium (CM) of AML-12 were used to treat BMDMs (Figure S8). The mRNA expression of *CCR2* was significantly up-regulated in AML-12-LPS-CM treated *Piezo1^fl/fl^* BMDMs compared with* Piezo1^ΔLysM^* BMDMs (Figure 3G). Similarly, FCM displayed that the population of CCR2^+^ cells and its median fluorescence intensity (MFI) were notably increased in AML-12-LPS-CM treated* Piezo1^fl/fl^* BMDMs (Figure 3H). Taken together, Piezo1 could moderate macrophage infiltration *in vivo* and *in vitro*.

### Myeloid-specific *Piezo1* deficiency decreases inflammation and M1 polarization in fibrotic livers

**Figure 4:**
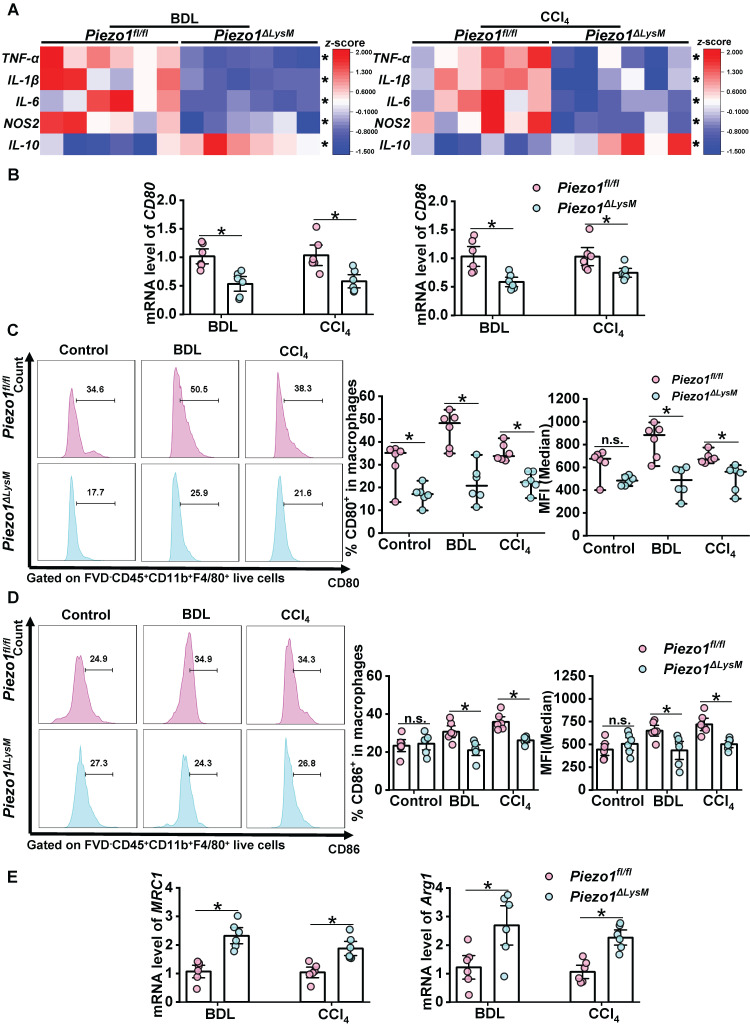
** Myeloid-specific *Piezo1* deficiency decreases inflammation and M1 polarization in fibrotic livers. (A)** Relative mRNA expression levels of *TNF-α*, *IL-6* , *IL-1β*, *NOS2* and *IL-10* in liver tissues of *Piezo1^fl/fl^* and *Piezo1^ΔLysM^* mice were measured. Heatmap shown the z-score of gene levels. **(B)** *CD80* and* CD86* mRNA levels in liver tissues of *Piezo1^fl/fl^* and *Piezo1^ΔLysM^* mice were measured. The percentage of hepatic **(C)** CD80*^+^* macrophages and **(D)** CD86*^+^* macrophages were shown by Histogram (left panels), their proportion (middle panels) and MFI (right panels) were quantified by FCM. **(E)** *MRC1* and *Arg1* mRNA levels in liver tissues of *Piezo1^fl/fl^* and *Piezo1^ΔLysM^* mice were measured. Data are presented as mean ± S. E. M.; n = 6 for each group; *P < 0.05.

Next, we investigated the potential role of myeloid Piezo1 in the regulation of inflammation within fibrotic livers. RT-qPCR showed higher expression of *TNF-α*, *IL-1β*, *IL-6* and *NOS2*, while lower expression of *IL-10* in fibrotic livers of *Piezo1^fl/fl^* mice than *Piezo1^ΔLysM^* littermates (Figure 4A). M1 macrophages, regarded as pro-inflammatory phenotype and characterized by high expression of CD80 and CD86, participate in hepatic inflammation and fibrosis. The mRNA levels of *CD80* and *CD86* in fibrotic livers of *Piezo1^fl/fl^* mice were significantly higher than* Piezo1^ΔLysM^* littermates (Figure 4B). Similarly, FCM showed that the population of CD80^+^ and CD86^+^ macrophages and its MFI were significantly increased in fibrotic livers of *Piezo1^fl/fl^* mice compared with* Piezo1^ΔLysM^* littermates (Fig 4C-D). In addition, higher mRNA expression of *MRC1* and *Arg1* were detected in hepatic fibrotic *Piezo1^ΔLysM^* mice than *Piezo1^fl/fl^* littermates (Figure 4E). Herein, RT-qPCR and FCM analysis revealed that myeloid-specific Piezo1 regulates inflammation and M1 polarization in fibrotic livers.

### Activation of myeloid Piezo1 enhances cathepsin S (CTSS) expression and activity *in vitro* and *in vivo*

**Figure 5:**
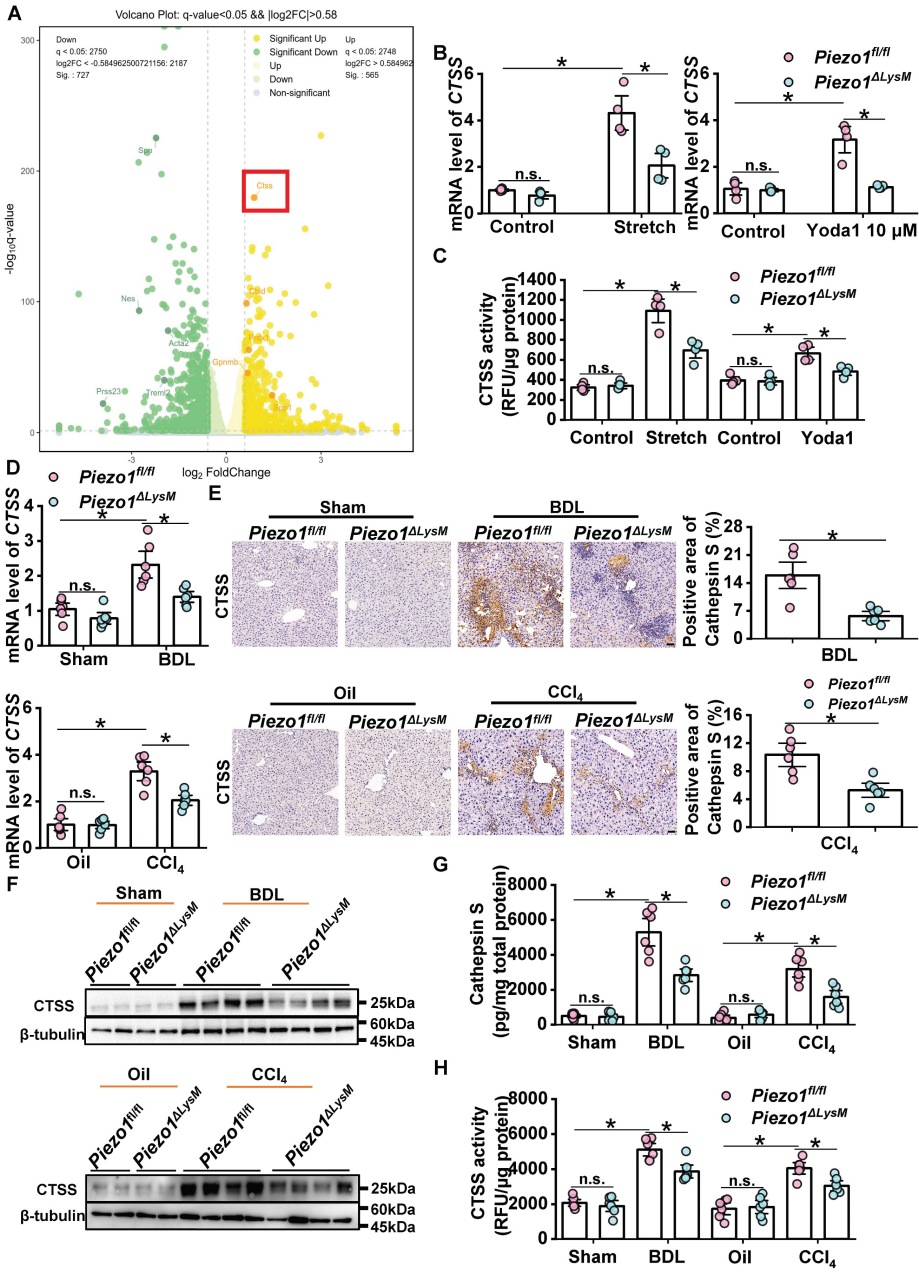
** Myeloid Piezo1 activation enhances cathepsin S expression and activity *in vitro* and *vivo*. (A)** Volcano plots were shown based on the result of RNA-seq. Green and yellow color represents low and high expression values, respectively.** (B)** Relative mRNA expression of *CTSS* and **(C)** CTSS activity in *Piezo1^fl/fl^* and* Piezo1^ΔLysM^* BMDMs were measured. **(D)** Relative mRNA expression of *CTSS*, **(E)** immunochemistry staining of CTSS, **(F)** western blot images with CTSS,** (G)** the protein expression of CTSS detected by ELISA and **(H)** CTSS activity in liver tissues of *Piezo1^fl/fl^* and* Piezo1^ΔLysM^* mice were measured. Scale bar, 100 μm. Data are presented as mean ± S. E. M.; mice samples: n = 6 for each group; cell samples: n = 4 for each group; western blot: n = 4 for each group; *P < 0.05.

As unveiled by our research group and corroborated by others, Piezo1 could mediate Ca^2+^ activity in macrophages 16, 26, 37. To investigate the mechanisms of macrophage Piezo1 influences liver fibrosis, BMDMs from *Piezo1^fl/fl^* mice were treated with mechanical stretch for RNA-seq experiments. The heatmap showed differentially expressed genes in stretch and control *Piezo1^fl/fl^* BMDMs (Figure S9). The volcano plot exhibited that *CTSS* was significantly up-regulated in mechanical stretched BMDMs compared with control BMDMs (Figure 5A). We found that the mRNA expression of *CTSS* markedly amplified in *Piezo1^fl/fl^* BMDMs treated with mechanical stretch or Piezo1 specific agonist Yoda1 relative to that in controls, but the growth trend in *Piezo1^ΔLysM^* BMDMs was significantly restrained (Figure 5B). Similarly, the same trend was obtained by the measurement of CTSS activity (Figure 5C).

Consequently, we investigated whether myeloid-specific *Piezo1* knockout suppresses CTSS expression and activity in livers of BDL-operated and CCl_4_-injected mice. The mRNA expression of *CTSS* was significantly decreased in fibrotic livers of *Piezo1^ΔLysM^* mice compared with* Piezo1^fl/fl^* littermates (Figure 5D). Hepatic CTSS expression, which was measured by immunochemistry staining, western blot and Elisa, was markedly increased in BDL-operated and CCl_4_-injected* Piezo1^fl/fl^* mice compared with *Piezo1^ΔLysM^* littermates (Figure 5E-G). The same trend was observed with CTSS activity (Figure 5H). These data suggested that macrophage Piezo1 regulates CTSS expression and activity.

### Macrophage Piezo1 regulates proliferation and activation of T cells in fibrotic livers

**Figure 6:**
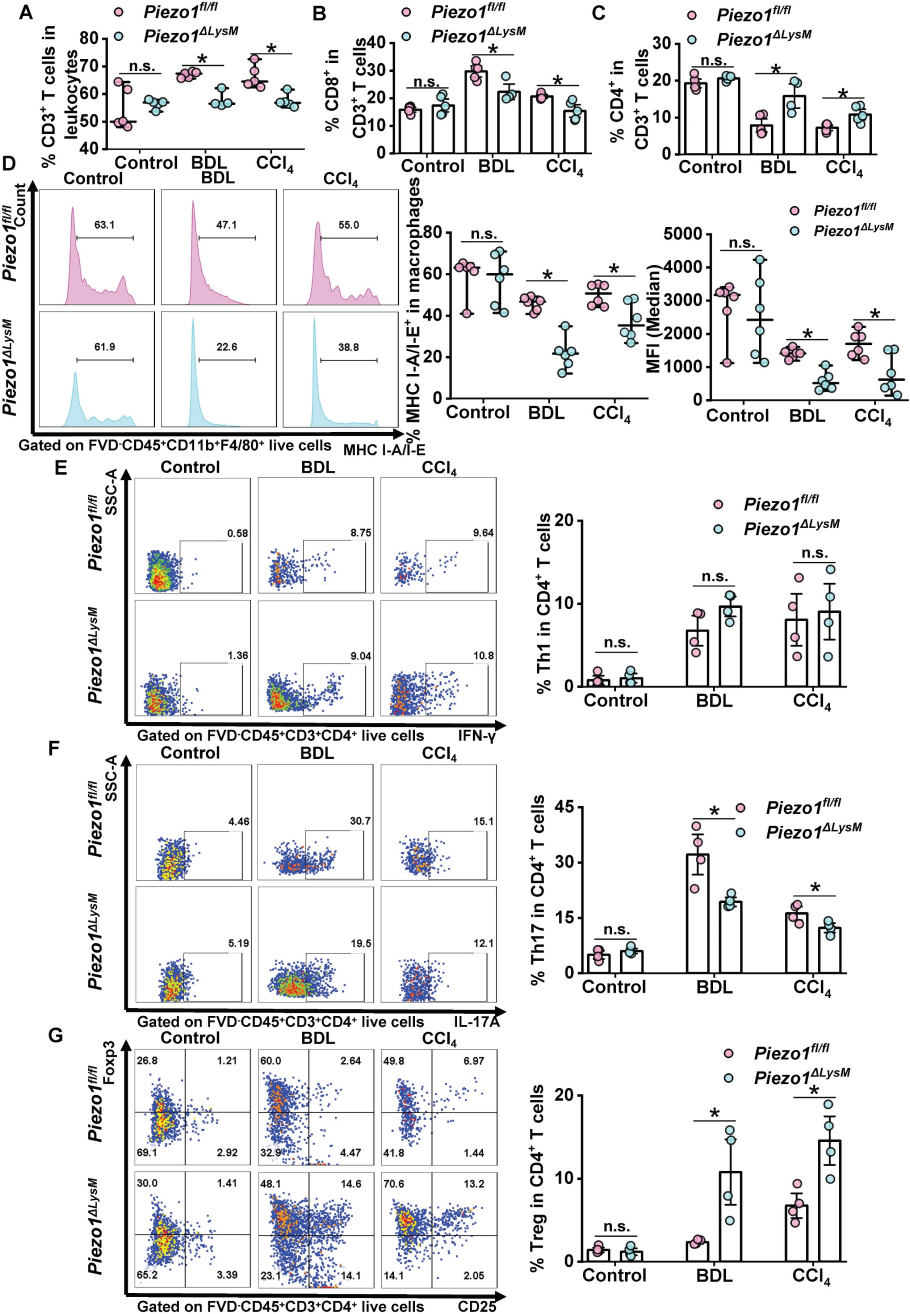
** Macrophage Piezo1 regulates T cells proliferation and activation in fibrotic livers.** The percentage of hepatic **(A)** CD3*^+^* T cells (FVD*^-^*CD45*^+^*CD3*^+^*), **(B)** CD8^+^ T cells (FVD*^-^*CD45*^+^*CD3*^+^*CD8*^+^*) and **(C)** CD4^+^ T cells (FVD*^-^*CD45*^+^*CD3*^+^*CD4*^+^*) were quantified in liver tissues of *Piezo1^fl/fl^* and* Piezo1^ΔLysM^* mice by FCM. **(D)** The percentage of hepatic MHC I-A/I-E^+^ macrophages shown by Histogram (left panels), their proportion (middle panels) and MFI (right panels) were quantified by FCM. The population of **(E)** Th1 cells (FVD*^-^*CD45*^+^*CD3*^+^*CD4*^+^*IFN-γ*^+^*), **(F)** Th17 cells (FVD*^-^*CD45*^+^*CD3*^+^*CD4*^+^*IL-17A*^+^*) and **(G)** Treg cells (FVD*^-^*CD45*^+^*CD3*^+^*CD4*^+^*CD25*^+^*Foxp3*^+^*) were quantified in livers of *Piezo1^fl/fl^* and* Piezo1^ΔLysM^* mice by FCM. Data are presented as mean ± S. E. M.; **(A-C)** n = 4-5 for each group; **(D)** n = 6 for each group; **(E-G)** n = 4 for each group; *P < 0.05.

As mentioned above, myeloid Piezo1 regulates macrophage infiltration, M1 polarization and CTSS expression in fibrotic livers. No alterations were observed in the proportion of hepatic and splenic dendritic cells (DCs) in fibrotic *Piezo1^fl/fl^* and *Piezo1^ΔLysM^* mice (Figure S10). Next, we explored whether myeloid-specific *Piezo1* has a role in T cell proliferation and activation during liver fibrosis. FCM showed the proportion of CD3^+^ T cells and CD8^+^ T cells were decreased, whereas the number of CD4^+^ T cells were markedly increased in fibrotic livers of Piezo1*^ΔLysM^* mice compared with Piezo1*^fl/fl^* littermates (Figure 6A-C, Figure S11). CTSS regulates MHC-II signaling pathway to control CD4^+^ T cell activation 30. As antigen processing cells, macrophages regulate the T cell function 38, 39. FCM showed lower population of MHC I-A/I-E^+^ macrophages in fibrotic livers of *Piezo1^ΔLysM^* mice than Piezo1*^fl/fl^* littermates (Figure 6D). No alterations were found in the proportion of Th1 cells in fibrotic livers between *Piezo1^ΔLysM^* and *Piezo1^fl/fl^* littermates (Figure 6E). The population of Th17 cells was significantly expanded, while Treg cells exhibited an opposite trend in hepatic fibrotic* Piezo1^fl/fl^* mice compared with *Piezo1^ΔLysM^* mice (Figure 6F-G). In spleen, the frequency of CD4^+^ T cells was markedly reduced in fibrotic *Piezo1^ΔLysM^* mice. The percentage of CD8^+^ T cells showed a reduction after undergoing BDL surgery in *Piezo1^ΔLysM^* mice. However, no significant changes were observed in the percentage of CD3^+^ T cells (Figure S12A-C). Thus, our data suggested that macrophage Piezo1 could alter proliferation and activation of T cells in fibrotic livers.

### CTSS regulates inflammatory response mediated by Piezo1 in BMDMs

**Figure 7:**
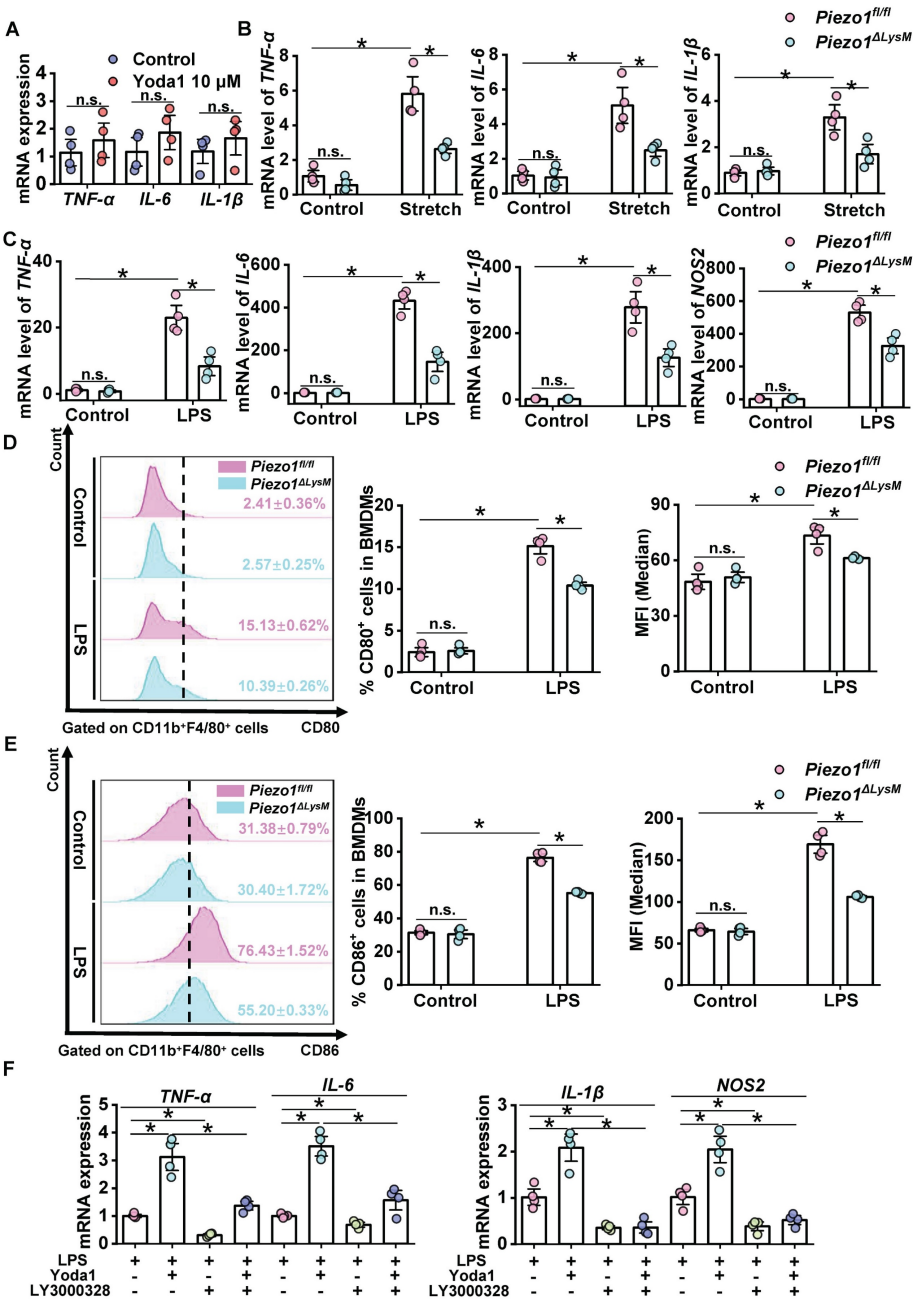
** CTSS regulates inflammatory response mediated by Piezo1 in BMDMs. (A)** Relative mRNA expression of *TNF-α, IL-6* and *IL-1β* in BMDMs from C57BL/6J mice were quantified.** (B)** Relative mRNA expression of *TNF-α, IL-6* and* IL-1β* in *Piezo1^fl/fl^* and* Piezo1^ΔLysM^* BMDMs were measured. **(C)** Relative mRNA expression of *TNF-α*,* IL-6*,* IL-1β* and *NOS2* in *Piezo1^fl/fl^* and* Piezo1^ΔLysM^* BMDMs were measured. **(D)** Representative FCM Histogram showed CD80^+^ cells (left panels), the percentage of CD80*^+^* cells (middle panels) and the MFI (right panels) in *Piezo1^fl/fl^* and* Piezo1^ΔLysM^* BMDMs. **(E)** Representative FCM Histogram showed CD86*^+^* cells (left panels), the percentage of CD86*^+^* cells (middle panels) and the MFI (right panels) in *Piezo1^fl/fl^* and* Piezo1^ΔLysM^* BMDMs. **(F)** Relative mRNA expression of *TNF-α*,* IL-6*,* IL-1β* and *NOS2* in *Piezo1^fl/fl^* BMDMs were measured. Data are presented as mean ± S. E. M.; n = 4 for each group; *P < 0.05.

A recent study revealed that macrophage activation can be regulated by Piezo1 26. To explore functions of Piezo1 in macrophage activation, we used Yoda1 to treat BMDMs from C57BL/6J mice. The mRNA expression levels of *IL6*,* TNF-α* and* IL1β* in Yoda1-stimulated BMDMs were slightly elevated but no significant changes compared with controls (Figure 7A). Then, *Piezo1^fl/fl^* and* Piezo1^ΔLysM^* BMDMs were stimulated with mechanical stretch. The mRNA levels of *IL1β*,* TNF-α* and* IL6* in *Piezo1^fl/fl^* BMDMs were markedly higher than those in *Piezo1^ΔLysM^* BMDMs (Figure 7B). Our data indicated that Piezo1 could control macrophage activation.

In order to induce macrophage inflammatory response, *Piezo1^fl/fl^* and *Piezo1^ΔLysM^* BMDMs were stimulated with LPS. The mRNA expression of *TNF-α*,* IL6*,* IL1β* and* NOS2* from LPS-treated *Piezo1^fl/fl^* BMDMs were markedly up-regulated compared with LPS-treated *Piezo1^ΔLysM^* controls (Figure 7C). Similarly, the proportion of CD80^+^ and CD86^+^ cells and MFI were significantly higher in LPS-stimulated* Piezo1^fl/fl^* BMDMs than *Piezo1^ΔLysM^* controls (Figure 7D-E). In addition, compared with LPS-stimulated* Piezo1^fl/fl^* BMDMs, higher mRNA levels of *TNF-α*,* IL6*,* IL1β* and* NOS2* were detected in LPS and Yoda1-stimulated *Piezo1^fl/fl^* BMDMs. However, inhibition of CTSS blocked the inflammatory response in *Piezo1^fl/fl^* BMDMs induced by LPS or both LPS and Yoda1 (Figure 7F). These data indicated that Piezo1 regulates BMDMs inflammatory response.

### Myeloid-specific *Piezo1* deletion inhibits activation of human hepatic stellate cells (LX-2)

As shown above, we found macrophage Piezo1 deletion attenuates its inflammatory activation *in vitro* assays. LPS-stimulated *Piezo1^ΔLysM^* BMDMs significantly reduced LX-2 activation compared with those in *Piezo1^fl/fl^* BMDMs (Figure S13). To investigate whether specific activation of Piezo1 in BMDMs affects activation of hepatic stellate cells (HSCs), LX-2 cells were treated with the clarified supernatant of mechanical stretch or Yoda1-stimulated BMDMs for 24h. Immunostaining of α-SMA, which is a marker of myofibroblast, showed LX-2 cells were remarkedly elongated from the CM of *Piezo1^fl/fl^* BMDMs treated with mechanical stretch or Yoda1, but not from that of *Piezo1^ΔLysM^* controls (Figure S14A, C). The gene expression of *Col1*, *Col3, α-SMA*, *vimentin* and *TGF-β* were increased in LX-2 cells stimulated by the supernatant from stretch or Yoda1-treated* Piezo1^fl/fl^* BMDMs relative to those in *Piezo1^ΔLysM^* controls (Figure S14B, D). Herein, macrophage Piezo1 could regulate fibroblast activation during liver fibrosis.

### Macrophage CTSS secretion is mediated by Piezo1/Calpain/LAMP1 axis

**Figure 8:**
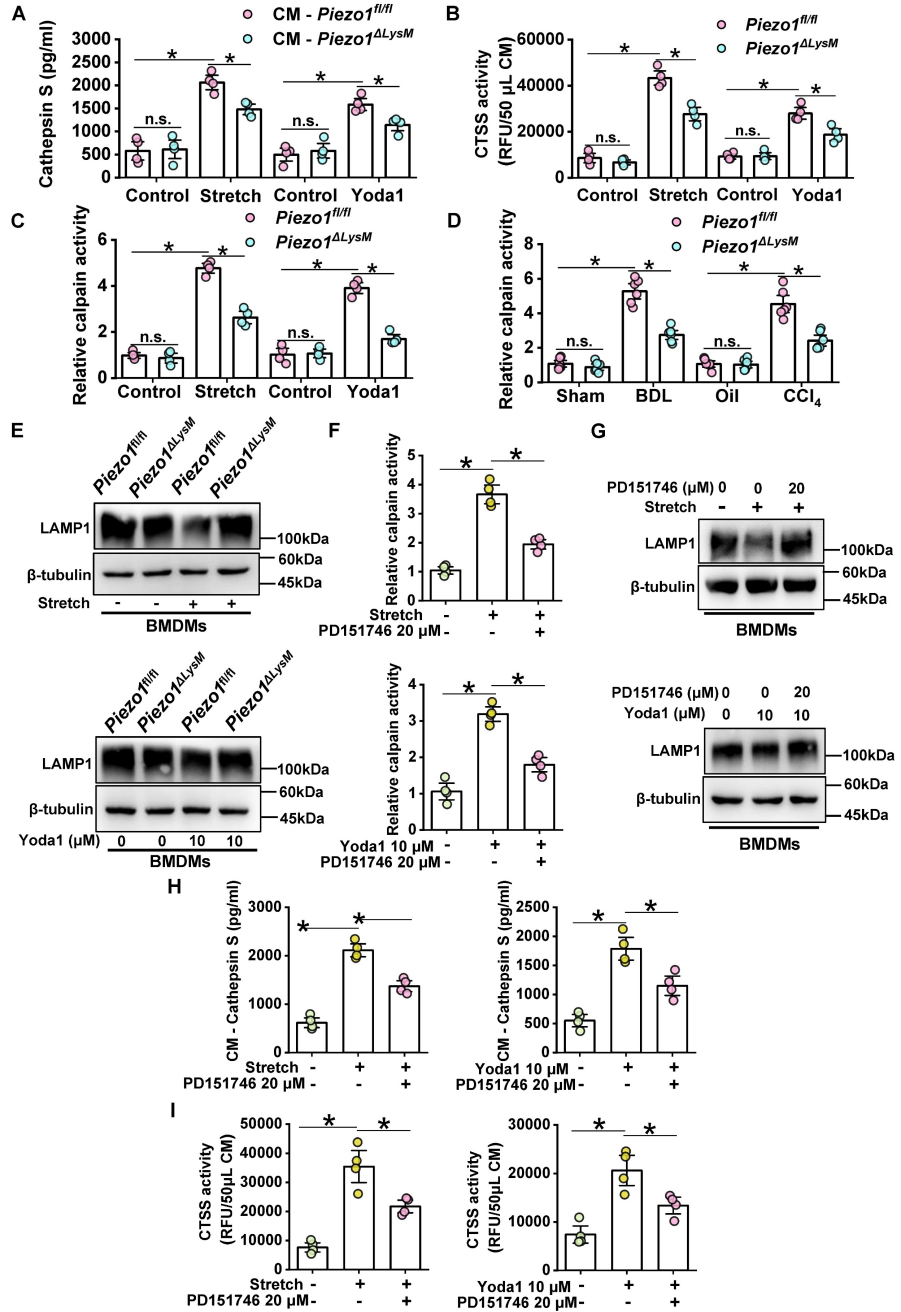
** Macrophage CTSS secretion is mediated by Piezo1/Calpain/LAMP1 axis. (A)** The protein level of CTSS and **(B)** its activity were measured in CM (conditioned culture medium) collected from *Piezo1^fl/fl^* and* Piezo1^ΔLysM^* BMDMs. **(C)** Calpain activity was detected in *Piezo1^fl/fl^* and* Piezo1^ΔLysM^* BMDMs. **(D)** Calpain activity in livers of* Piezo1^fl/fl^* and* Piezo1^ΔLysM^* mice were detected.** (E)** Western blot images of LAMP1 in *Piezo1^fl/fl^* and* Piezo1^ΔLysM^* BMDMs were shown. **(F)** Calpain activity and **(G)** western blot images of LAMP1 were measured in mechanical stretch or Yoda1-stimulated *Piezo1^fl/fl^* BMDMs treated with or without PD151746. **(H)** The protein level and **(I)** activity of CTSS were measured in CM collected from mechanical stretch or Yoda1-stimulated *Piezo1^fl/fl^* BMDMs treated with or without PD151746. Data are presented as mean ± S. E. M.; mice samples: n = 6 for each group; cell samples: n = 4 for each group; western blot: n = 3 for each group; *P < 0.05.

Some studies have revealed CTSS secretion to extracellular space could induce ECM remodeling 34, 40, 41. We examined the secretion of CTSS in cell supernatants from BMDMs. Increased expression and enzymatic activity of CTSS were observed in the supernatant of mechanical stretch and Yoda1-stimulated* Piezo1^fl/fl^* BMDMs. Conversely, this growth trend was blunted in *Piezo1^ΔLysM^* BMDMs, indicating a regulatory role of Piezo1 in the secretion of CTSS in BMDMs (Figure 8A-B).

Calpain activity can be regulated by Piezo1 in endothelial cells and macrophages 16, 42. Indeed, after treating with stretch or Yoda1, calpain activity was markedly higher in *Piezo1^fl/fl^* BMDMs, whereas it was inhibited in *Piezo1^ΔLysM^* BMDMs (Figure 8C). Similarly, calpain activity was remarkable higher in fibrotic livers of *Piezo1^fl/fl^* mice than *Piezo1^ΔLysM^* littermates (Figure 8D). The gene expression of *CAPN1* and *CAPN2* were markedly increased in fibrotic livers of *Piezo1^fl/fl^* mice compared with *Piezo1^ΔLysM^* littermates (Figure S15). Activation of calpain could degrade LAMP1 to change lysosomal permeability and secrete Cathepsins 43. Indeed, western blot and immunofluorescence staining showed LAMP1 expression was significantly reduced in *Piezo1^fl/fl^* BMDMs treated with stretch or Yoda1 compared with controls, but it was reversed in *Piezo1^ΔLysM^* BMDMs (Figure 8E, Figure S16A-B).

To further investigate its underlying mechanisms, BMDMs isolated from *Piezo1^fl/fl^* mice were treated with stretch or Yoda1 and calpain inhibitor PD151746. We found that PD151746 could significantly inhibited calpain activity induced by stretch or Yoda1 in BMDMs (Figure 8F). In addition, western blot showed calpain inhibition reversed the degradation of LAMP1 induced by stretch or Yoda1 (Figure 8G). Furthermore, the secretion and enzymatic activity of CTSS in the supernatant were markedly reduced in calpain inhibition (Figure 8H-I). These data indicated that Piezo1 could regulate LAMP1 via calpain activity to control CTSS secretion.

## Discussion

**Figure 9:**
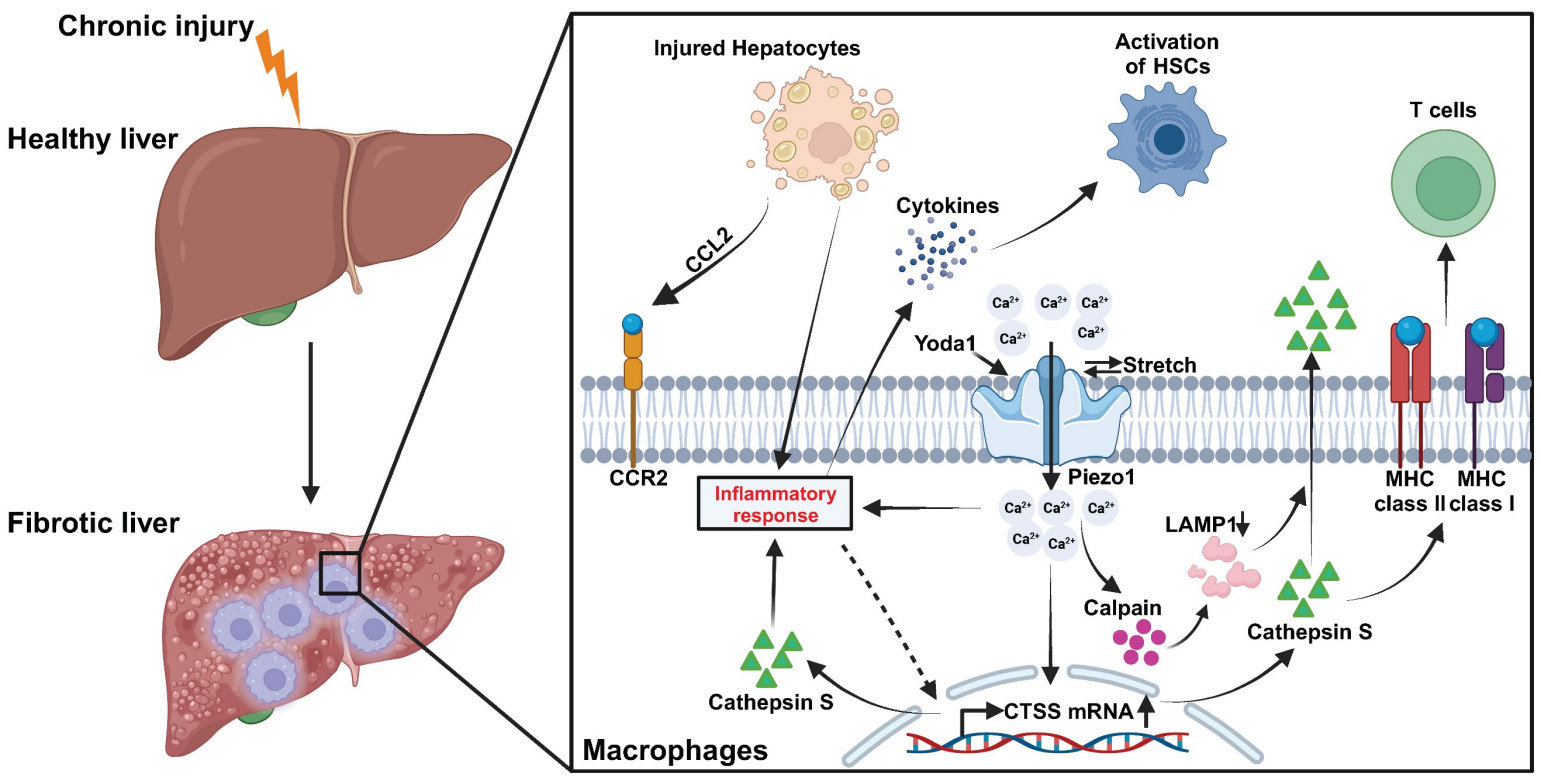
Graphic illustration on Piezo1 reprogrammed macrophage activation and CTSS function to promotes HSCs activation and regulates T cell activity during liver fibrosis.

The pivotal role of macrophage Piezo1 in liver fibrosis is substantiated in our investigation. First, a positive correlation is observed between increased macrophage Piezo1 expression and liver fibrosis in both human and mouse samples. Second, the absence of myeloid-specific *Piezo1* regulates macrophage infiltration, restricts M1 polarization, and diminishes the inflammatory response in fibrotic livers. Third, activation of macrophage Piezo1 boosts the expression and activity of CTSS through Ca^2+^ influx. Fourth, macrophage Piezo1 governs T cell activity and HSCs activation during liver fibrosis. Fifth, Piezo1 activation partially modulates macrophage inflammation by affecting CTSS activity. Finally, the secretion of macrophage CTSS is mediated by the Piezo1/calpain/LAMP1 axis. Collectively, these findings underscore the critical contribution of macrophage Piezo1 as a pivotal factor in liver fibrosis (Figure 9).

It's widely reported that Piezo1 expression is correlated with progression of fibrosis in different mice model 15, 16, 18. In the development of liver fibrosis, the gradual deposition of ECM increases compressional and tensional forces around cells. Excessive ECM induces fibrotic zones (30 kPa) much harder than healthy liver tissues (about 5 kPa) 11, 12. On the other hand, an uncontrolled and sustained wound healing response results in the excessive accumulation of ECM, leading to the destruction of normal hepatic lobules, the formation of false lobules, and the induction of portal hypertension. 9. All these can be sensed by Piezo1, which is essential for cells to sense mechanical forces 13. Our data showed that higher Piezo1 expression was observed in human and mice fibrotic livers. Therefore, it's reasonable to consider that expression of Piezo1 may be upregulated in cells upon liver injury.

Macrophages are regarded as crucial regulators in all stages of liver fibrosis, including inflammation, fibrosis progression and its resolution 44. Macrophage Piezo1 is highly expressed compared with other mechanosensitive cation proteins 19, 26. Increased Piezo1 expression in macrophage cell line when cultured in higher stiffness hydrogel or stimulated by LPS was reported 26. Consistent with previous study, we found higher expression of Piezo1 in mechanical stretch or LPS-stimulated BMDMs compared with controls *in vitro*. Macrophages regulate their differentiation when they are recruited to injury areas or fibrotic zones 45. Both external mechanical forces (compression, stiffness, shear stress etc.) and internal actin polymerization induce a change of lipid bilayer tension, that activates Piezo1 directly and permits Ca^2+^ influx 26, 46, 47. The activation of Piezo1 stimulated by biomechanical stretch promotes nuclear factor kappa-light-chain-enhancer of activated B cells (NF-κB) phosphorylation, which is essential for macrophage activation 26. In addition, Piezo1 mediates macrophage infiltration by regulating CCL2 and CCR2 expression and controlling Notch signaling pathway in BMDMs 16. Significant upregulation of Piezo1 expression in macrophages was detected in both human and mouse fibrotic liver tissues. Additionally, the knockout of myeloid *Piezo1* was found to suppress hepatic fibrosis by inhibiting macrophage recruitment, reducing inflammation, and impeding M1 polarization. Furthermore, through in vitro assays, we verified that the activation of Piezo1 promoted macrophage inflammatory response.

The interaction between macrophages and HSCs in liver fibrosis have been widely proved 48, 49. Besides macrophages, other immune cells, such as neutrophils 50, DCs 51, T cells 52-54, etc., participate in development of hepatic fibrosis partially by crosstalk with HSCs. Piezo1 plays a role on innate and adaptive immune response 19, 55. Our data showed that myeloid-specific *Piezo1* regulated immune cells infiltration. However, how myeloid *Piezo1* influence immune microenvironment and activation of HSCs during liver fibrosis needs further examinations.

Overexpression of CTSS has been reported to play crucial roles in fibrosis 34, 56, 57. The expression of CTSS is regulated by activation of transcription factor EB (TFEB) 58, inflammatory cytokines 59, or others 60, 61. Increased intracellular Ca^2+^ concentration could induce TFEB activation to promote lysosomal biogenesis 62. Our study found the activation of Piezo1 promoted CTSS synthesis in macrophages. Intracellular CTSS is regarded as an important factor in antigen processing by regulating T cell activity via MHC-I and MHC-II signaling pathway 30. In addition, CTSS regulates secretion of inflammatory cytokines and expression of MHC-II and CD80 in macrophages 63. Our findings suggested that the inhibition of CTSS partially mitigated macrophage inflammation induced by Piezo1 activation. Moreover, myeloid-specific *Piezo1* played a regulatory role in the subset of MHC-II^+^ macrophages and T cell activity in fibrotic livers. It is plausible that the interaction between CD4^+^ T cells and macrophages is mediated by Piezo1/CTSS axis.

For extracellular CTSS, we noticed expression and activity of CTSS were increased in supernatant from BMDMs stimulated by Yoda1 or mechanical stretch. Calpain activation induced by Piezo1-madiated Ca^2+^ influx has been reported 42. In addition, calpain cleaves LAMP1 and LAMP2 to destroy the integrity of lysosomes, then soluble hydrolases such as CTSS are released from lysosomes to extracellular space 43, 64, 65. We found macrophage Piezo1 activation regulated LAMP1 degradation by enhanced calpain activity. Furthermore, inhibition of calpain activity significantly limited Yoda1 or stretch-induced macrophage degradation of LAMP1 and secretion of CTSS. CTSS has been confirmed to directly enhance the ability of collagen synthesis in fibroblast 34. In addition, decorin, a natural inhibitor of TGF-β, plays a protective role in liver fibrosis and can be degraded by CTSS 66, 67. Currently, proving that extracellular CTSS/TGF-β signaling pathway mediated by macrophage Piezo1 requires further examinations.

In conclusion, our study revealed the key roles of macrophage Piezo1 in progression of hepatic fibrosis. Myeloid-specific Piezo1 regulates macrophage infiltration, inflammation, M1 polarization as well as synthesis and secretion of CTSS, which promotes HSCs activation and T cell activity during liver fibrosis. Our research provides the evidences that Piezo1 channels are a crucial factor in regulating macrophage inflammatory response and pharmacological intervention of Piezo1 provide new strategies to treat chronic liver diseases.

## Supplementary Material

Supplementary materials and methods, figures and tables.

## Abbreviations

ALT: alanine aminotransferase
Arg1: arginase 1
AST: aspartate aminotransferase
α-SMA: alpha-smooth muscle actin
BA: biliary atresia
BDL: bile duct ligation
BMDMs: bone marrow-derived macrophages
CCL2: C-C Motif chemokine ligand 2, also known as MCP-1: monocyte chemoattractant protein-1
CCl_4_: carbon tetrachloride
CCR2: C-C chemokine receptor-2
CM: conditioned culture medium
Col1: collagen I
Col3: collagen III
Ctrl: control
CTSS: cathepsin S
ECM: extracellular matrix
EMT: epithelial-mesenchymal transition
FCM: flow cytometry
FN: fibronectin
H&E: hematoxylin-eosin
HBV: hepatitis B
HCC: hepatocellular carcinoma
HSCs: hepatic stellate cells
IL: interleukin
LAMP1: lysosome-associated membrane protein-1
LPS: lipopolysaccharide
MFI: median fluorescence intensity
MRC1: mannose receptor C type 1
NOS: nitric oxide synthase
PBC: primary biliary cirrhosis
RFU: relative fluorescence unit
RNA-seq: RNA-sequencing
RT-qPCR: Real Time Quantitative PCR
SR: Sirius red
TGF-β: transforming growth factor-β
Th cell: T helper cell
TNF: tumor necrosis factor
Treg: regulatory T cell
Vim: vimentin

## References

[1] Bataller R, Brenner DA (2005). Liver fibrosis. J Clin Invest.

[2] Friedman SL (2003). Liver fibrosis - from bench to bedside. J Hepatol.

[3] Lo RC, Kim H (2017). Histopathological evaluation of liver fibrosis and cirrhosis regression. Clin Mol Hepatol.

[4] Tacke F (2017). Targeting hepatic macrophages to treat liver diseases. J Hepatol.

[5] Duffield JS, Forbes SJ, Constandinou CM, Clay S, Partolina M, Vuthoori S (2005). Selective depletion of macrophages reveals distinct, opposing roles during liver injury and repair. J Clin Invest.

[6] Holt MP, Cheng L, Ju C (2008). Identification and characterization of infiltrating macrophages in acetaminophen-induced liver injury. J Leukoc Biol.

[7] Pradere JP, Kluwe J, De Minicis S, Jiao JJ, Gwak GY, Dapito DH (2013). Hepatic macrophages but not dendritic cells contribute to liver fibrosis by promoting the survival of activated hepatic stellate cells in mice. Hepatology.

[8] Ramachandran P, Pellicoro A, Vernon MA, Boulter L, Aucott RL, Ali A (2012). Differential Ly-6C expression identifies the recruited macrophage phenotype, which orchestrates the regression of murine liver fibrosis. Proc Natl Acad Sci U S A.

[9] Boyer-Diaz Z, Aristu-Zabalza P, Andrés-Rozas M, Robert C, Ortega-Ribera M, Fernández-Iglesias A (2021). Pan-PPAR agonist lanifibranor improves portal hypertension and hepatic fibrosis in experimental advanced chronic liver disease. J Hepatol.

[10] Leung VY, Shen J, Wong VW, Abrigo J, Wong GL, Chim AM (2013). Quantitative elastography of liver fibrosis and spleen stiffness in chronic hepatitis B carriers: comparison of shear-wave elastography and transient elastography with liver biopsy correlation. Radiology.

[11] Sandrin L, Fourquet B, Hasquenoph JM, Yon S, Fournier C, Mal F (2003). Transient elastography: a new noninvasive method for assessment of hepatic fibrosis. Ultrasound Med Biol.

[12] Ziol M, Handra-Luca A, Kettaneh A, Christidis C, Mal F, Kazemi F (2005). Noninvasive assessment of liver fibrosis by measurement of stiffness in patients with chronic hepatitis C. Hepatology.

[13] Coste B, Mathur J, Schmidt M, Earley TJ, Ranade S, Petrus MJ (2010). Piezo1 and Piezo2 are essential components of distinct mechanically activated cation channels. Science.

[14] Volkers L, Mechioukhi Y, Coste B (2015). Piezo channels: from structure to function. Pflugers Arch.

[15] Zhao X, Kong Y, Liang B, Xu J, Lin Y, Zhou N (2022). Mechanosensitive Piezo1 channels mediate renal fibrosis. JCI Insight.

[16] He Y, Deng B, Liu S, Luo S, Ning Y, Pan X (2022). Myeloid Piezo1 Deletion Protects Renal Fibrosis by Restraining Macrophage Infiltration and Activation. Hypertension.

[17] Blythe NM, Muraki K, Ludlow MJ, Stylianidis V, Gilbert HTJ, Evans EL (2019). Mechanically activated Piezo1 channels of cardiac fibroblasts stimulate p38 mitogen-activated protein kinase activity and interleukin-6 secretion. J Biol Chem.

[18] Zhang Y, Su SA, Li W, Ma Y, Shen J, Wang Y (2021). Piezo1-Mediated Mechanotransduction Promotes Cardiac Hypertrophy by Impairing Calcium Homeostasis to Activate Calpain/Calcineurin Signaling. Hypertension.

[19] Solis AG, Bielecki P, Steach HR, Sharma L, Harman CCD, Yun S (2019). Mechanosensation of cyclical force by PIEZO1 is essential for innate immunity. Nature.

[20] Huang JQ, Zhang H, Guo XW, Lu Y, Wang SN, Cheng B (2021). Mechanically Activated Calcium Channel PIEZO1 Modulates Radiation-Induced Epithelial-Mesenchymal Transition by Forming a Positive Feedback With TGF-beta1. Front Mol Biosci.

[21] Swain SM, Romac JM, Vigna SR, Liddle RA (2022). Piezo1-mediated stellate cell activation causes pressure-induced pancreatic fibrosis in mice. JCI Insight.

[22] Hu J, Chen Q, Zhu H, Hou L, Liu W, Yang Q (2022). Microglial Piezo1 senses Abeta fibril stiffness to restrict Alzheimer's disease. Neuron.

[23] Liu L, Yu H, Zhao H, Wu Z, Long Y, Zhang J (2020). Matrix-transmitted paratensile signaling enables myofibroblast-fibroblast cross talk in fibrosis expansion. Proc Natl Acad Sci U S A.

[24] Hsieh JY, Smith TD, Meli VS, Tran TN, Botvinick EL, Liu WF (2017). Differential regulation of macrophage inflammatory activation by fibrin and fibrinogen. Acta Biomater.

[25] Cha BH, Shin SR, Leijten J, Li YC, Singh S, Liu JC (2017). Integrin-Mediated Interactions Control Macrophage Polarization in 3D Hydrogels. Adv Healthc Mater.

[26] Atcha H, Jairaman A, Holt JR, Meli VS, Nagalla RR, Veerasubramanian PK (2021). Mechanically activated ion channel Piezo1 modulates macrophage polarization and stiffness sensing. Nat Commun.

[27] Ma S, Dubin AE, Zhang Y, Mousavi SAR, Wang Y, Coombs AM (2021). A role of PIEZO1 in iron metabolism in mice and humans. Cell.

[28] Hilscher MB, Sehrawat T, Arab JP, Zeng Z, Gao J, Liu M (2019). Mechanical Stretch Increases Expression of CXCL1 in Liver Sinusoidal Endothelial Cells to Recruit Neutrophils, Generate Sinusoidal Microthombi, and Promote Portal Hypertension. Gastroenterology.

[29] Brown R, Nath S, Lora A, Samaha G, Elgamal Z, Kaiser R (2020). Cathepsin S: investigating an old player in lung disease pathogenesis, comorbidities, and potential therapeutics. Respir Res.

[30] Dheilly E, Battistello E, Katanayeva N, Sungalee S, Michaux J, Duns G (2020). Cathepsin S Regulates Antigen Processing and T Cell Activity in Non-Hodgkin Lymphoma. Cancer cell.

[31] Vizovišek M, Fonović M, Turk B (2019). Cysteine cathepsins in extracellular matrix remodeling: Extracellular matrix degradation and beyond. Matrix Biol.

[32] de Mingo A, de Gregorio E, Moles A, Tarrats N, Tutusaus A, Colell A (2016). Cysteine cathepsins control hepatic NF-kappaB-dependent inflammation via sirtuin-1 regulation. Cell Death Dis.

[33] Clark AK, Wodarski R, Guida F, Sasso O, Malcangio M (2010). Cathepsin S release from primary cultured microglia is regulated by the P2X7 receptor. Glia.

[34] Xie Y, Fontenot L, Chupina Estrada A, Nelson B, Wang J, Shih DQ (2022). Elafin Reverses Intestinal Fibrosis by Inhibiting Cathepsin S-Mediated Protease-Activated Receptor 2. Cell Mol Gastroenterol Hepatol.

[35] Baeck C, Wehr A, Karlmark KR, Heymann F, Vucur M, Gassler N (2012). Pharmacological inhibition of the chemokine CCL2 (MCP-1) diminishes liver macrophage infiltration and steatohepatitis in chronic hepatic injury. Gut.

[36] Sun X, Wu J, Liu L, Chen Y, Tang Y, Liu S (2022). Transcriptional switch of hepatocytes initiates macrophage recruitment and T-cell suppression in endotoxemia. J Hepatol.

[37] Pan X, Wan R, Wang Y, Liu S, He Y, Deng B (2022). Inhibition of chemically and mechanically activated Piezo1 channels as a mechanism for ameliorating atherosclerosis with salvianolic acid B. Br J Pharmacol.

[38] Blazar BR, Murphy WJ, Abedi M (2012). Advances in graft-versus-host disease biology and therapy. Nat Rev Immunol.

[39] Zhang X, Xu H, Yu J, Cui J, Chen Z, Li Y (2023). Immune Regulation of the Liver Through the PCSK9/CD36 Pathway During Heart Transplant Rejection. Circulation.

[40] Chen H, Wang J, Xiang MX, Lin Y, He A, Jin CN (2013). Cathepsin S-mediated fibroblast trans-differentiation contributes to left ventricular remodelling after myocardial infarction. Cardiovasc Res.

[41] Small DM, Brown RR, Doherty DF, Abladey A, Zhou-Suckow Z, Delaney RJ (2019). Targeting of cathepsin S reduces cystic fibrosis-like lung disease. Eur Respir J.

[42] Li J, Hou B, Tumova S, Muraki K, Bruns A, Ludlow MJ (2014). Piezo1 integration of vascular architecture with physiological force. Nature.

[43] Villalpando Rodriguez G, Torriglia A (2013). Calpain 1 induce lysosomal permeabilization by cleavage of lysosomal associated membrane protein 2. Biochim Biophys Acta.

[44] Campana L, Iredale JP (2017). Regression of Liver Fibrosis. Semin Liver Dis.

[45] Krenkel O, Tacke F (2017). Liver macrophages in tissue homeostasis and disease. Nat Rev Immunol.

[46] Ellefsen KL, Holt JR, Chang AC, Nourse JL, Arulmoli J, Mekhdjian AH (2019). Myosin-II mediated traction forces evoke localized Piezo1-dependent Ca(2+) flickers. Commun Biol.

[47] Lewis AH, Grandl J (2015). Mechanical sensitivity of Piezo1 ion channels can be tuned by cellular membrane tension. Elife.

[48] Li S, Zhou B, Xue M, Zhu J, Tong G, Fan J (2023). Macrophage-specific FGF12 promotes liver fibrosis progression in mice. Hepatology.

[49] Rao J, Wang H, Ni M, Wang Z, Wang Z, Wei S (2022). FSTL1 promotes liver fibrosis by reprogramming macrophage function through modulating the intracellular function of PKM2. Gut.

[50] Zhou Z, Xu MJ, Cai Y, Wang W, Jiang JX, Varga ZV (2018). Neutrophil-Hepatic Stellate Cell Interactions Promote Fibrosis in Experimental Steatohepatitis. Cell Mol Gastroenterol Hepatol.

[51] Connolly MK, Bedrosian AS, Mallen-St Clair J, Mitchell AP, Ibrahim J, Stroud A (2009). In liver fibrosis, dendritic cells govern hepatic inflammation in mice via TNF-alpha. J Clin Invest.

[52] Li H, Ding P, Peng B, Ming YZ (2021). Cross-talk between hepatic stellate cells and T lymphocytes in liver fibrosis. Hepatobiliary Pancreat Dis Int.

[53] Koda Y, Teratani T, Chu PS, Hagihara Y, Mikami Y, Harada Y (2021). CD8(+) tissue-resident memory T cells promote liver fibrosis resolution by inducing apoptosis of hepatic stellate cells. Nat Commun.

[54] Zhou Y, Zhang H, Yao Y, Zhang X, Guan Y, Zheng F (2022). CD4(+) T cell activation and inflammation in NASH-related fibrosis. Front Immunol.

[55] Wang Y, Yang H, Jia A, Wang Y, Yang Q, Dong Y (2022). Dendritic cell Piezo1 directs the differentiation of T(H)1 and T(reg) cells in cancer. Elife.

[56] Weldon S, McNally P, McAuley DF, Oglesby IK, Wohlford-Lenane CL, Bartlett JA (2014). miR-31 dysregulation in cystic fibrosis airways contributes to increased pulmonary cathepsin S production. Am J Respir Crit Care Med.

[57] Yao X, Cheng F, Yu W, Rao T, Li W, Zhao S (2019). Cathepsin S regulates renal fibrosis in mouse models of mild and severe hydronephrosis. Mol Med Rep.

[58] Ni H, Xu S, Chen H, Dai Q (2020). Nicotine Modulates CTSS (Cathepsin S) Synthesis and Secretion Through Regulating the Autophagy-Lysosomal Machinery in Atherosclerosis. Arterioscler Thromb Vasc Biol.

[59] Caglic D, Repnik U, Jedeszko C, Kosec G, Miniejew C, Kindermann M (2013). The proinflammatory cytokines interleukin-1alpha and tumor necrosis factor alpha promote the expression and secretion of proteolytically active cathepsin S from human chondrocytes. Biol Chem.

[60] Gautam J, Bae YK, Kim JA (2017). Up-regulation of cathepsin S expression by HSP90 and 5-HT(7) receptor-dependent serotonin signaling correlates with triple negativity of human breast cancer. Breast Cancer Res Treat.

[61] Yan D, Wang HW, Bowman RL, Joyce JA (2016). STAT3 and STAT6 Signaling Pathways Synergize to Promote Cathepsin Secretion from Macrophages via IRE1alpha Activation. Cell Rep.

[62] Nakamura S, Shigeyama S, Minami S, Shima T, Akayama S, Matsuda T (2020). LC3 lipidation is essential for TFEB activation during the lysosomal damage response to kidney injury. Nat Cell Biol.

[63] Thanei S, Theron M, Silva AP, Reis B, Branco L, Schirmbeck L (2017). Cathepsin S inhibition suppresses autoimmune-triggered inflammatory responses in macrophages. Biochem Pharmacol.

[64] Peintner L, Venkatraman A, Waeldin A, Hofherr A, Busch T, Voronov A (2021). Loss of PKD1/polycystin-1 impairs lysosomal activity in a CAPN (calpain)-dependent manner. Autophagy.

[65] Eskelinen EL (2006). Roles of LAMP-1 and LAMP-2 in lysosome biogenesis and autophagy. Mol Aspects Med.

[66] Baghy K, Iozzo RV, Kovalszky I (2012). Decorin-TGFβ axis in hepatic fibrosis and cirrhosis. J Histochem Cytochem.

[67] Kehlet SN, Bager CL, Willumsen N, Dasgupta B, Brodmerkel C, Curran M (2017). Cathepsin-S degraded decorin are elevated in fibrotic lung disorders - development and biological validation of a new serum biomarker. BMC Pulm Med.

