# Piezo1 specific deletion in macrophage protects the progression of liver fibrosis in mice: Erratum

**DOI:** 10.7150/thno.107640

**Published:** 2025-01-01

**Authors:** Shangfei Luo, Xiaoduo Zhao, Jintao Jiang, Bo Deng, Silin Liu, Honglin Xu, Qiaorui Tan, Yu'an Chen, Ziyan Zhang, Xianmei Pan, Rentao Wan, Xiaoting Chen, Youfen Yao, Jing Li

**Affiliations:** 1 Lingnan Medical Research Center, Guangzhou University of Chinese Medicine, Guangzhou, 510405, China.; 2 The First Affiliated Hospital, Guangzhou University of Chinese Medicine, Guangzhou, 510405, China.; 3 Department of Pathology, the First Affiliated Hospital of Zhejiang University School of Medicine, Hangzhou, 310006, China.; 4 The Second Affiliated Hospital of Guangzhou Medical University, Guangzhou Medical University, Guangzhou, 510260, China.; 5 Innovation Research Center, Shandong University of Chinese Medicine, Jinan, 250307, China.; 6 School of Biomedical Sciences, Faculty of Biological Sciences, University of Leeds, LS2 9JT, UK.

The authors regret that the original version of our paper contains an error in partially overlapping the images of the "sham" and "oil" groups in Figure 1D. This may be due to incorrect image selection during the initial draft writing and failure to carefully check the images before final version confirmation.

The correct Figure 1 appears below.

## Figures and Tables

**Figure 1:**
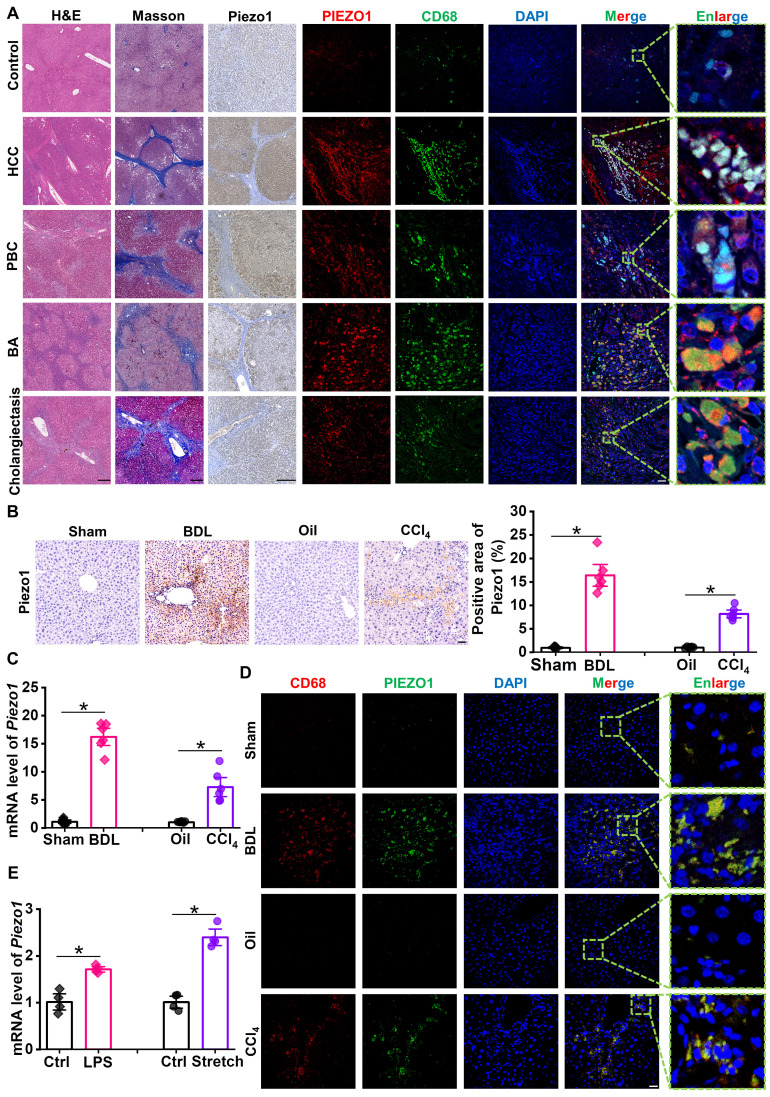
Expression of Piezo1 in macrophage has increased in fibrotic livers. **(A)** Representative images of H&E, Masson's, immunochemistry staining of Piezo1 and dual immunofluorescence staining with CD68 (green) and Piezo1 (red) in human liver samples. Scale bar, H&E and Masson's, 400 μm; immunochemistry, 200 μm; immunofluorescence, 50 μm, enlarge, 5.75 μm. (**B**) Representative images of Piezo1 staining and quantification of positive area in liver sections of C57BL/6J mice. Scale bar, 50 μm. **(C)** Relative mRNA expression of *Piezo1* in liver tissues of C57BL/6J mice. **(D)** Representative images of dual immunofluorescence staining with CD68 (red) and Piezo1 (green) in liver sections of C57BL/6J mice. Scale bar, 25 μm; enlarge, 5 μm. (**E**) Relative mRNA expression of *Piezo1* in BMDMs isolated from C57BL/6J mice. Data are presented as mean ± S. E. M.; Human samples: control (n = 3), HBV-related HCC (n = 4), PBC (n = 5), BA (n = 4), cholangiectasis (n = 4); mice samples (n = 6 for each group); cell samples (n = 4 for each group). *P < 0.05.

