# Supplementary material for: Piezo1 specific deletion in macrophage protects the progression of liver fibrosis in mice
Source: Theranostics. 2023 Oct 2;13(15):5418–34. doi: 10.7150/thno.86103 (PMC10614683; doi:10.7150/thno.86103)
Supplement: Supplementary file 1 — Supplementary materials and methods, figures and tables. [file thnov13p5418s1.pdf]

Data Supplement

Piezo1 specific deletion in macrophage protects the progression of liver fibrosis in mice

Shangfei Luo#, **Xiaoduo Zhao**#, Jintao Jiang, Bo Deng, Silin Liu, Honglin Xu, Qiaorui Tan, Yu'an Chen, Ziyan Zhang, Xianmei Pan, Rentao Wan, Xiaoting Chen, Youfen Yao, Jing Li*

#These authors contribute equally

*Corresponding authors.

Supplement Materials

This file includes:

- **Methods**
- **Table S1. The detailed information of patients.**
- **Table S2. Sequences of the primers used for mice genotype.**
- **Table S3. Sequences of the primers used for RT-PCR.**
- **Table S4. List of antibodies.**
- **Figure S1. The specificity of the Piezo1 antibody was measured by immunofluorescence staining.**
- **Figure S2. Liver fibrosis was attenuated in BDL-operated and CCl_4-injected *Piezo1*$^{+/-}$ mice.**
- **Figure S3. Validation of Piezo1 deletion in BMDMs.**
- **Figure S4. Piezo1 expression is increased in fibrotic livers.**
- **Figure S5. Myeloid-specific *Piezo1* deletion attenuates liver fibrosis.**
- **Figure S6. Myeloid-specific *Piezo1* doesn't influence neutrophils infiltration in murine fibrotic livers.**
- **Figure S7. Myeloid-specific *Piezo1* doesn't affect the number of splenic myeloid cells.**
- **Figure S8. Schematic diagram of BMDMs treated with conditioned culture medium collected from AML-12.**
- **Figure S9. Heatmap exhibited that differentially expressed genes in stretch and control *Piezo1*$^{fl/fl}$ BMDMs.**
- **Figure S10. Myeloid-specific Piezo1 doesn't affect hepatic and splenic dendritic cells infiltration in mice.**
- **Figure S11. Liver T cells were shown by FCM gating strategy.**
- **Figure S12. Splenic T cells were shown by FCM gating strategy.**
- **Figure S13. Myeloid-specific *Piezo1* regulates activation of human hepatic stellate cells (LX-2) through impairing macrophage inflammatory response.**
- **Figure S14. Myeloid-specific *Piezo1* deletion inhibits activation of LX-2.**
- **Figure S15. Myeloid-specific *Piezo1* regulates calpain activity in murine fibrotic livers.**

- **Figure S16. Activation of Piezo1 controls degradation of LAMP1.**

Methods

Animals

Myeloid-specific *Piezo1* deletion mice (*Piezo1*$^{\triangle LysM}$, C57BL/6J background) were generated using the Cre-loxp system. *Piezo1*$^{fl/fl}$ (No. 029213, from Jackson Laboratories, C57BL/6J background) and LysM-Cre mice (No. 004781, from Jackson Laboratories, C57BL/6J background) were crossed to obtain *Piezo1* $^{\triangle LysM}$ mice and Piezo1$^{fl/fl}$ mice for littermate controls [1]. *Piezo1*$^{+/-}$ (heterozygous) and *Piezo1*$^{+/+}$ (wildtype) mice were described previously [2]. Mice were housed in a specified pathogen-free environment at temperature-controlled and 12 h light/12 h dark cycles with normal diet. All animal experiments were approved by the Guangzhou University of Chinese Medicine Animal Ethics Committee (approval no. 2017030617). Mouse genotyping was performed by PCR. PCR primers were shown on shown on Table S2. About 6-8 weeks male mice were selected for experiments, and all mice were genotyped before and after the experiments with PCR primers.

Human Samples

All pathologic evaluations and data for patients were approved by Clinical Research Ethics Committee of the First Affiliated Hospital, Zhejiang University School of Medicine (Acceptance number: IIT20200767A). All recruited patients have informed consents and were strictly de-identified and anonymized.

Mouse model of liver fibrosis

Murine hepatic fibrosis was induced by bile duct ligation (BDL) and carbon tetrachloride (CCl_4) injection.

For BDL-induced liver fibrosis, the details of the BDL surgery were reported [3]. Briefly, mice were anesthetized by inhalation of 2% isoflurane, then the abdomen was opened through midline. The common bile duct was separated carefully and ligated with 5-0 operative suture at two different locations. The gallbladder was not removed. The sham-operated control mice were suffered the same process but without ligation. The mice were sacrificed 14 days after the surgery.

For CCl_4-induced liver fibrosis, mice were intraperitoneal injection of 2 mL/kg CCl_4 (Macklin, C805329, China) dissolved in olive oil (Macklin, O815211, China; 1:4 volume ratio) three times a week for 4 weeks, control mice were injected with equal volume of olive oil. The mice were killed 24 h after the final injection.

Cell culture

L-929 cells (mouse fibroblast cells) and LX-2 (human hepatic stellate cells) were purchased from the American Type Culture Collection (ATCC, USA) and cultured in DMEM (Gibco, USA) medium containing 10% fetal bovine serum (FBS, Gibco, USA) and 1% penicillin and streptomycin (P/S, Gibco, USA) at 37 °C, 5% CO_2 incubator.

AML-12 (mouse hepatocyte cell line) was purchased from Shanghai Institute of Biochemistry and Cell Biology (Shanghai, China) and maintained in DMEM/F12 (Gibco, USA) with 10% FBS, 40 ng/mL dexamethasone, 1x ITS Liquid Media Supplement (Sigma-Aldrich, I3146, USA) and 1% P/S.

Induction of bone marrow-derived macrophages (BMDMs)

BMDMs were isolated from 6-8 week old C57BL/6J, $Piezo1^{\triangle LysM}$ and $Piezo1^{fl/fl}$ male mice. Briefly, mice were sacrificed and cut off the foot, then removed the muscle attached to the femur and tibia swiftly. After soaking in 75% ethanol for 5 min, each bone was severed proximal to each joint. Bone marrow was flushed using DMEM, then cell suspension was collected. Cell suspension was filtered with 40 μm cell strainer (Falcon, USA) and centrifuged at 1,000 rpm for 5 min at room temperature with no brake. Cell precipitation was resuspended in complete medium containing 30% L-929 conditioned culture medium, 20% FBS, 1% P/S, 1% glutamine (Gibco, USA) and DMEM, then plated in 100mm^2 Petri dish (Corning, USA). After 3 days of culture, 5 mL freshly complete medium was added. Macrophages incubated in medium were fully differentiated for 7 days at 37 °C, 5% CO_2 incubator.

Cell treatment

AML-12 treatment was performed as follows: lipopolysaccharide (LPS, Pepro Tech, USA,

100 ng/mL for 12 h) for injured state, then cell supernatants were collected, clarified and treated BMDMs for 6 h.

BMDMs treatment was performed as follows: LPS (100 ng/mL for 6 h,) for inflammatory response, Yoda1 (10 μM for 6 h, Tocris, UK) or mechanical stretch application for Piezo1 activation, and PD151746 treatment (20 μM, Selleck, USA) for inhibition of calpain activity. The cell supernatants were collected and clarified. For inhibition of CTSS, BMDMs were treated with LY3000328 (20 μM, MedChemExpress, HY-15533, China) overnight.

LX-2 were stimulated by conditioned culture medium collected from BMDMs for 24 h.

Intracellular Ca^{2+} measurements

About 90% density BMDMs were plated in a clear 96-well plate. After removing the culture medium, cells were incubated at 37 °C for 1 h with a loading solution containing 2 μM Fura-2-AM (Thermo Fisher Scientific, USA) and 0.01% pluronic acid (Thermo Fisher Scientific, USA) in a standard solution, which comprised (in mM) 5 KCl, 130 NaCl, 1.2 $MgCl_2$, 1.5 $CaCl_2$, 8 D-glucose, and 10 HEPES, and was titrated to pH 7.4 with NaOH. Intracellular Ca^{2+} signal was measured using a 96-well plate reader (FlexStation 3, Molecular Devices, USA). Changes(Δ) of intracellular Ca^{2+} concentration were determined by the Fura-2 ratio calculation of emission intensity at 510 nm after 340 and 380 nm excitation wavelengths.

Biochemical assays.

Serum alanine aminotransferase (ALT) and aspartate aminotransferase (AST) were measured using AST/GOT Assay Kit (Nanjing Jiancheng Bioengineering Institute, C010-2-1, China) and ALT/GPT Assay Kit (Nanjing Jiancheng Bioengineering Institute, C009-2-1, China) following the manufacturer's directions.

Histology and immunohistochemistry

For Hematoxylin & eosin (H&E), Masson's trichrome, and Sirius red staining, tissue slices (4 μm) of 4% paraformaldehyde-fixed, paraffin-embedded liver tissues were deparaffinized, hydrated, then stained with commercial Hematoxylin & eosin staining kit (Servicebio, G1076,

China), Masson's trichrome staining kit (Servicebio, G1006, China) and Sirius red staining kit (Phygene, PH1098, China) according to the manufacturer's instructions.

For immunohistochemistry staining (IHC), briefly, 4 μm paraffin-embedded liver slices were treated following by the steps of the conventional dewaxing, hydration and antigen retrieval. Endogenous peroxidase was blocked by 3% H_2O_2 at room temperature for 10 min and nonspecific binding sites were attenuated by a mixture solution containing 0.1% Triton X-100 and 10% donkey serum in PBS for 2 h at room temperature. The slices were incubated with primary antibodies (shown in Table S4) overnight at 4 ℃, then stained with HRP antibodies (shown in Table S4) for 1 h at room temperature. The remaining steps were followed by the protocol of GTvision immunohistochemical Kit (Gene Tech, GK500710, Shanghai, China).

Images of mice slices were acquired on Pannoramic MIDI (3D HISTECH, Europe). At least 3 different fields in each liver sample were imaged. The quantification of Masson's trichrome, Sirius red staining and IHC staining were performed using Image J. Images of human slices were captured by Nikon microscope (Nikon, Tokyo, Japan).

Immunofluorescence staining

For mice samples, liver tissues were submerged with 4% paraformaldehyde. The samples were lowered to the bottom after 15% and 30% sucrose gradient dehydrated. Frozen OCT-embedded liver samples were sliced into 6 μm sections. For human liver samples, 4 μm paraffin-embedded liver slices were treated following by the steps of the conventional dewaxing, hydration and antigen retrieval. Tissues were permeabilized with 0.1% Triton X-100, blocked with 10% Donkey serum for 1 h at room temperature and washed in PBS. Then samples were incubated with appropriate primary antibodies (shown in Table S4) overnight at 4 ℃. Then secondary antibodies with fluorescence (shown in Table S4) were applied at room temperature for 1-2 h. All samples were stained with DAPI (Sigma-Aldrich, USA), and coverslips were applied with fluoromount-G (SouthernBiotech, USA). At least 3 different fields in each sample were imaged.

For cell samples, cells were fixed by 4% paraformaldehyde, then the procedure were performed as described above. At least 3 different fields in each sample were imaged.

All pictures were captured by A Leica confocal microscope (M165 FC). Quantification was

performed using Image J.

RNA isolation, cDNA synthesis and RT-qPCR.

Total RNA from liver tissues and cell samples was extracted using TRIzol (Accurate Biology, AG21102, China) followed by the manufacturer's protocol. The concentration of total RNA was measured by NanoDrop (Thermo Fisher Scientific, USA). Then cDNA was synthesized by RNA using Evo M-MLV Reverse Transcription Kit (Accurate Biology, AG11705, China). RT-qPCR was performed on a QuantStudio™ 5 Real-Time PCR Instrument (Applied Biosystems Thermo Fisher Scientific, USA) using SYBR® Green Pro Taq HS Premixed qPCR kit (Accurate Biology, AG11701, China) and two-step reaction procedures were performed according to the standard protocol: 95 °C for 30 s, followed by 39 cycles of 95 °C for 5 s, 60 °C for 40 s, then melting (melting curve stage) at 78 °C for 5 s and 95°C for 1 s. The expression of mRNA was normalized with 18s and calculated using 2-△△Ct. The primers are listed in the primer list (shown on Table S3).

Isolation of immune cells.

The isolation of immune cells from liver for flow cytometry was described previously [4]. Briefly, mice were deep anesthetized and perfused with 0.9% saline by heart. The liver was taken out carefully and cut into small pieces, which were digested by HBSS (1x) supplemented with 0.01% type IV collagenase (Sigma, USA), 5 mM HEPEs and 0.5 mM $CaCl_2$ for 30 min at 37 °C and cold HBSS (1x) supplemented with 2% FBS were added to terminate the digestion. Cell suspension was collected, then filtered through a 70 μm cell strainer. After centrifuging at 50 xg for 10 min, the cell supernatant was obtained and centrifuged at 500 xg for 5 min. Cell precipitation was resuspended in cold HBSS (1x) and RBC lysis was using red cell lysis buffer (Biosharp, China) for 3 min on ice, then centrifuged at 500 xg for 5 min. After washing with HBSS (1x), the cell pellet was resuspended in HBSS (1x) and purified using 33% percoll (Cytiva, USA) density gradient and centrifuged at 800 xg for 25 min. The cells were collected and resuspended in HBSS (1x) to a final density of 1×10^6 cells per 100 μL.

For isolation of immune cells from spleen, spleen was ground on top of a 70 μm cell strainer,

then centrifuged at 400 xg for 5 min at 4°C. Then cell precipitation was resuspended in cold HBSS (1x) and RBC lysis was using red cell lysis buffer (Biosharp, China) for 3 min on ice, then centrifuged at 400 xg for 5 min. The cell pellet was resuspended in HBSS (1x) to a final density of 1×10^6 cells per 100 μL.

Flow cytometry

To detect cell surface marker, tissue samples were blocked with Fc Block for 30 min on ice, then stained with antibodies for 30 min on ice (shown in the list of antibodies, Table S4) and washed. To detect intracellular cytokines in $CD4^+$ T cells (gated on $FVD^-CD45^+CD3^+CD4^+$) isolated from liver, cells were incubated in 1640 medium supplemented with 10% FBS, 1% P/S, 30 ng/mL phorbol 12-myristate 13-acetate (PMA, sigma, P1585, USA), 1 μg/ml ionomycin (Macklin, I838446, China), and 1.7 μg/mL monensin (MedChemExpress, 17090-79-8, China) for 6 h at 37 °C, 5% CO_2 incubator. Cells were first blocked with Fc Block for 30 min on ice, then labeled with cell surface markers (shown in the list of antibodies, Table S4). Cells were washed, fixed and permeabilized using True-Nuclear™ Transcription Factor Buffer Set (Biolegend, 424401, USA). Antibodies (shown in Table S4) were used to stain IFN-γ, IL-17A and Foxp3. Samples were analyzed on a BD LSRFortessa X-20 flow cytometer (BD Bioscience, USA).

For BMDM samples, cells were digested and washed twice with HBSS (1x), followed by staining with antibodies (shown in the list of antibodies, Table S4) and analyzed by using BD LSRFortessa™ (BD Bioscience, USA).

All sample data analyses were performed using FlowJo_V10.6.2.

Calpain activity measurement

Calpain activity of tissues and cells was measured by calpain activity assay kit (Abcam, ab65308, UK). Liver tissues were harvested, washed by cold PBS and homogenized in the extraction buffer. The tissues were then centrifuged at 10,000 xg for 10 min at 4 °C. To measure protein concentrations, BCA protein assay kit was used followed by diluting 100 μg samples with extraction buffer to the volume of 85 μL and then transferred to a 96-well plate. Subsequently, 5 μL calpain substrate and 10 μL reaction buffer were added to each assay well

and incubated in the dark at 37 °C for 1 h. Absorbance was measured on a plate reader at an excitation of 400 nm and emission at 505 nm. For BMDM calpain activity measurement, cells were collected in the supplied extraction buffer, and assays were performed as described above.

Mechanical stretch experiments

The Flexcell FX-5000 Tension System (Flexcell International Corporation, USA) was used for BMDMs mechanical stretch assays. Briefly, BMDMs were seeded about 90% density per well onto type I collagen-coated Bioflex 6-well plates (Flexcell International Corporation, USA) and let the cells settled down to the membrane for 24 h. The BioFlex plates were placed on vacuum-based loading docks of the Flexcell FX-5000 apparatus and cultured in a 37 °C humidified incubator at 5% CO_2 with 10% elongation at 1 Hz for 24 h. Cells used as the control experiments were growing on the Bioflex 6-well plates without stretching.

RNA sequencing analysis.

Total RNA samples of *Piezo1*$^{fl/fl}$ BMDMs treated with mechanical stretch and control were extracted using the TRIzol reagent (Invitrogen, CA, USA) according to the manufacturer's protocol. RNA purity and quantification were evaluated using the NanoDrop 2000 spectrophotometer (Thermo Scientific, USA). RNA integrity was assessed using the Agilent 2100 Bioanalyzer (Agilent Technologies, Santa Clara, CA, USA). Then the libraries were constructed using VAHTS Universal V6 RNA-seq Library Prep Kit according to the manufacturer's instructions. The transcriptome sequencing and analysis were conducted by OE Biotech Co., Ltd. (Shanghai, China).

The libraries were sequenced on a llumina Novaseq 6000 platform. Gene differentially expression analysis was performed using the DESeq2. Q-value < 0.05 and fold change (FC) > 1.5 was set as the threshold for differential expression gene.

Western Blot

By using the lysis buffer (Cell Signaling Technology, USA) containing1 mM PMSF (Keygen Biotech, China) and protease and phosphatase inhibitors (Keygen Biotech, China), the total

proteins from liver tissues and cell samples were extracted. The samples were vortexed gently every 10 min while allowing the lysed samples on ice for half an hour and followed by centrifugation at 12,000 xg at 4 °C for 15 min. The supernatants were collected and concentration was measured using the Pierce BCA Protein Assay Kit (Thermo Fisher Scientific, USA). 5x SDS-PAGE Sample Loading Buffer (Beyotime, P0015, China) was added to supernatant, then boiled at 100 °C for 10 min. 30 μg protein lysates were uniformed, run on 8-12% SDS-PAGE gels and transferred onto PVDF membranes (Millipore, USA). The membranes were blocked with 5% skim milk (Bio-Rad, USA) followed by incubation with primary antibodies (shown in the list of antibodies, Table S4) overnight before washing the membranes with PBST and incubated with a secondary antibody (shown in the list of antibodies, Table S4) for 60 min at RT. Blots were visualized using a ChemiScope system (CLiNX, China) after incubation with ECL western blot detection reagent (Millipore, USA).

Enzyme-linked immunosorbent assay (ELISA)

For liver samples, ELISA was performed using the protocol as described in the previous study [5]. For BMDMs, the supernatants were collected and centrifuged at 12,000 xg for 10 min. The CTSS concentration was measured using ELISA kits (Finetest, EM0701, China) following the recommended protocols.

CTSS activity measurement

CTSS activity in liver tissues, cells and cell supernatants were measured by Cathepsin S Activity Assay Kit (Fluorometric) (Abcam, ab65307, USA). First, liver tissue lysates and cell lysates centrifuged at 10 000 xg for 10 min at 4 °C to collect supernatants. The concentration was measured by BCA protein assay kit, then adjusted to 1mg/ml in 50 uL and transferred to a 96-well plate. CTSS substrate Ac-VVR-AFC (2 μL) and CTSS reaction buffer (50 μL) were added to each assay well, followed by incubation in the dark at 37 °C for 1 h. Relative fluorescence units were read with 400 nm excitation and 505 nm emission and calculated by subtracting background values. For measurement in cell supernatants, cell supernatants were collected and clarified, and assays were performed as described above.

Data analysis

All data were presented as mean ± S.E.M. Tests for statistical significance were performed using the SPSS 20 software and the graphs were managed by OriginPro 2018. Student's t-test or Mann-Whitney U test were used to compare the two data sets. One-way ANOVA followed by Bonferroni multiple comparison tests were performed in some data sets. P-value < 0.05 was considered statistically significant.

Reference

1. He Y, Deng B, Liu S, Luo S, Ning Y, Pan X, et al. Myeloid Piezo1 Deletion Protects Renal Fibrosis by Restraining Macrophage Infiltration and Activation. Hypertension. 2022; 79: 918-31.
2. Liu S, Xu X, Fang Z, Ning Y, Deng B, Pan X, et al. Piezo1 impairs hepatocellular tumor growth via deregulation of the MAPK-mediated YAP signaling pathway. Cell Calcium. 2021; 95: 102367.
3. Mukhopadhyay P, Rajesh M, Cao Z, Horváth B, Park O, Wang H, et al. Poly (ADP-ribose) polymerase-1 is a key mediator of liver inflammation and fibrosis. Hepatology. 2014; 59: 1998-2009.
4. Shi W, Wang Y, Zhang C, Jin H, Zeng Z, Wei L, et al. Isolation and purification of immune cells from the liver. Int Immunopharmacol. 2020; 85: 106632.
5. Loh Z, Fitzsimmons RL, Reid RC, Ramnath D, Clouston A, Gupta PK, et al. Inhibitors of class I histone deacetylases attenuate thioacetamide-induced liver fibrosis in mice by suppressing hepatic type 2 inflammation. Br J Pharmacol. 2019; 176: 3775-90.

Table S1. The detailed information of patients.

	Patient age	Serum total bilirubin (μmol/L)	Serum ALT (U/L)	Serum AST (U/L)	Serum albumin (g/L)
control (liver metastasis tumor)	54	5.8	21	13	46.9
	56	16.8	8	13	38.5
	71	13.5	11	20	34.9
primary biliary cirrhosis (PBC)	71	404.5	188	184	36.8
	52	613.1	70	68	29.3
	48	283.7	110	81	39.5
	44	133.3	49	212	34.5
	41	176	1184	676	37
hepatitis B cirrhosis-related hepatocellular carcinoma (HCC)	49	12.3	8	15	35.1
	42	26.2	73	35	41.2
	50	12.9	96	30	38.6
	55	7	29	29	72.7
biliary atresia (BA)	2	169.9	553	1825	30.3
	1	12.2	106	81	54.8
	3	395.8	60	121	39.7
	3m26d	3.9	12	28	41.3
cholangiectasis and hepatolithiasis	60	35.7	172	212	36
	57	19.1	170	44	40.5
	52	6.9	76	30	41.2
	52	19.6	92	89	39

Table S2. Sequences of the primers used for mice genotype.

Gene name	Species	primers
LysM (mutant)	Mouse	5’ - CCCAGAAATGCCAGATTACG - 3’
LysM (Common)	Mouse	5’ - CTTGGGCTGCCAGAATTTCTC - 3’
LysM (wild-type)	Mouse	5’ - TTACAGTCGGCCAGGCTGAC - 3’
Piezo1$^{+/-}$ *heterozygous (mutant)*	Mouse	5’ - TCCCAACCCCTTCCTCCTAC - 3’
Piezo1$^{+/-}$ *heterozygous (Common)*	Mouse	5’ - TGGCCCTGAAAGAAGTGAGT - 3’
Piezo1$^{+/-}$ *heterozygous (wild-type)*	Mouse	5’ - CATGAGGAATCACTGGGACA - 3’
LOXP-F	Mouse	5’ - GCCTAGATTCACCTGGCTTC - 3’
LOXP-R	Mouse	5’ - GCTCTTAACCATTGAGCCATCT - 3

Table S3. Sequences of the primers used for RT-PCR.

Gene name	Species	Forward primers	Reverse primers
18s	Mouse/human	TGGTTGCAAAGCTGAAACTTAAAG	AGTCAAATTAAGCCGCAGGC
Piezo1	Mouse	CACAAAGTACCGGGCG	AAAGTAAATGCACTTGACG
Collagen1	Mouse	GCTCCTCTTAGGGGCCACT	ATTGGGGACCCTTAGGCCAT
Collagen3	Mouse	CCACGAGGTGACAAAGGTGA	GCCAGGGAATCCTCGATGT
Vimentin	Mouse	CAGAGAGAG GAAGCCGAAAG	ATGCTGTTCCTGAATCTGGG
Fibronectin	Mouse	ATGTGGACCCCTCCTGATAGT	GCCCAGTGATTTCAGCAAAGG
α-SMA	Mouse	CCCAGACATCAGGGAGTAATGG	TCTATCGGATACTTCAGCGTCA
Snail1	Mouse	CACACGCTGCCTTGTGTCT	GGTCAGCAAAAGCACGGTT
TGF-β	Mouse	GAGCCCGAAGCGGACTACTA	TGGTTTTCTCATAGATGGCGTT
CCR2	Mouse	ATCCACGGCATACTATCAACATC	TCGTAGTCATACGGTGTGGTG
CCL2	Mouse	CCTGCTGCTACTCATTCACCA	ATTCCTTCTTGGGGTCAGCA
TNF-α	Mouse	ACGGCATGGATCTCAAAGAC	CGGACTCCGCAAAGTCTAAG
IL1β	Mouse	AGCTCTCCACCTCAATGGAC	GACAGGCTTGTGCTCTGCTT
IL6	Mouse	TCCATCCAGTTGCCTTCTTG	GGTCTGTTGGGAGTGGTATC
NOS2	Mouse	ACATCGACCCGTCCACAGTAT	CAGAGGGGTAGGCTTGTCTC
IL10	Mouse	AGCCTTATCGGAAATGATCCAGT	GGCCTTGTAGACACCTTGGT
MRC1	Mouse	CTCTGTTCAGCTATTGGACGC	CGGAATTTCTGGGATTCAGCTTC
Arg1	Mouse	CTCCAAGCCAAAGTCCTTAGAG	AGGAGCTGTCATTAGGGACATC
CD80	Mouse	TGGCTCTGCAAACACGGTTC	AGCAAATGTGCAAATCGTCCC
CD86	Mouse	TGTTTCCGTGGAGACGCAAG	TTGAGCCTTTGTAAATGGGCA
CTSS	Mouse	TACATTCAGCTCCCGTTTGGT	TCGTCATAGACACCGCTTTTGT
CAPN1	Mouse	ATGACAGAGGAGTTAATCACCCC	GGCTATGAGAAACCGGAGGG
CAPN2	Mouse	GGTCGCATGAGAGAGCCATC	CCCCGAGTTTTGCTGGAGTA
Collagen1	Human	CCCGGGTTTCAGAGACAACTTC	TCCACATGCTTTATTCCAGCAATC
Collagen3	Human	GGAGCTGGCTACTTCTCGC	GGGAACATCCTCCTTCAACAG

Vimentin	Human	TCTTCCAAACTTTTCCTCCC	AGTTTCGTTGATAACCTGTCC
α-SMA	Human	GGTGCTGTCTCTCTATGCCT	AAGGAATAGCCACGCTCAGT
TGF-β	Human	ACAGCAACAATTCCTGGCGA	CCGTTGATGTCCACTTGCAG

Table S4. List of antibodies

Antibodies	Origin	Product code	Dilution	Application
Anti-Piezo1 antibody	Proteintech	15939-1-AP	1:500	IHC
			1:100	IF
Anti-CD68 antibody	Abcam	Ab53444	1:400	IF
Anti-Collagen I antibody	Servicebio	GB11022-3	1:100	IF
Anti-Collagen III antibody	Servicebio	GB111629	1:200	IF
Anti-Vimentin antibody	abcam	ab92547	1:200	IHC
Anti-Fibronectin antibody	abcam	ab2413	1:200	IHC
Anti-α-SMA antibody	abcam	ab5694	1:200	IHC, IF
Anti-CD11b antibody	Novusbio	NB110-89474	1:300	IHC
Anti-F4/80 antibody	Servicebio	GB113373	1:500	IHC
Anti-Cathespin S antibody	Santa Cruz	SC-271619	1:100	IHC
			1:500	WB
Anti-LAMP1 antibody	abcam	ab208943	1:100	IF
			1:1000	WB
HRP anti-rabbit IgG	Servicebio	G1213	1:200	IHC
HRP anti-mouse IgG	Servicebio	G1214	1:200	IHC
Alexa Fluor™ 594 goat anti-rabbit IgG (H+L)	Invitrogen	A11012	1:800	IF
Alexa Fluor™ 488 goat anti-rabbit IgG (H+L)	Invitrogen	A11008	1:800	IF
Goat Anti-Rat IgG/S Alexa Fluor 647	Solarbio	K0032G	1:800	IF
Alexa Fluor™ 488 goat anti-rat IgG (H+L)	Invitrogen	A11006	1:800	IF
Purified anti-mouse CD16/32 Antibody	Biolegen	101301	1:100	FCM

	d			
Zombie Aqua Fixable Viability Dye	Biolegend	423101	1:100	FCM
APC/Cyanine7 anti-mouse/human CD11b Antibody	Biolegend	101226	1:100	FCM
PE anti-mouse F4/80 Antibody	Biolegend	123110	1:100	FCM
FITC anti-mouse CD45 Antibody	Biolegend	103108	1:100	FCM
Brilliant Violet 421™ anti-mouse Ly-6G Antibody	Biolegend	127627	1:100	FCM
PE anti-mouse CD11c Antibody	Biolegend	117307	1:100	FCM
Brilliant Violet 605 ™ anti-mouse CD80 Antibody	Biolegend	104729	1:100	FCM
Brilliant Violet 650™ anti-mouse CD86 Antibody	Biolegend	105036	1:100	FCM
Brilliant Violet 711™ anti-mouse I-A/I-E Antibody	Biolegend	107643	1:100	FCM
APC/Fire™ 750 anti-mouse CD3 Antibody	Biolegend	100248	1:100	FCM
Brilliant Violet 605™ anti-mouse CD4 Antibody	Biolegend	100547	1:100	FCM
Alexa Fluor® 700 anti-mouse CD8α Antibody	Biolegend	100730	1:100	FCM
Brilliant Violet 711™ anti-mouse IFN-γ Antibody	Biolegend	505835	1:100	FCM
Brilliant Violet 421™ anti-mouse IL-17A Antibody	Biolegend	506925	1:100	FCM
PE anti-mouse FOXP3 Antibody	Biolegend	126404	1:100	FCM
PE/Cyanine7 anti-mouse CD25 Antibody	Biolegend	102016	1:100	FCM
PE/Cyanine7 anti-mouse CD192 (CCR2) Antibody	Biolegend	150611	1:100	FCM
β-tubulin	CST	2128	1:1000	WB

HRP-conjugated anti-rabbit IgG	CST	7074s	1:5000	WB
HRP-conjugated anti-mouse IgG	CST	7076s	1:5000	WB

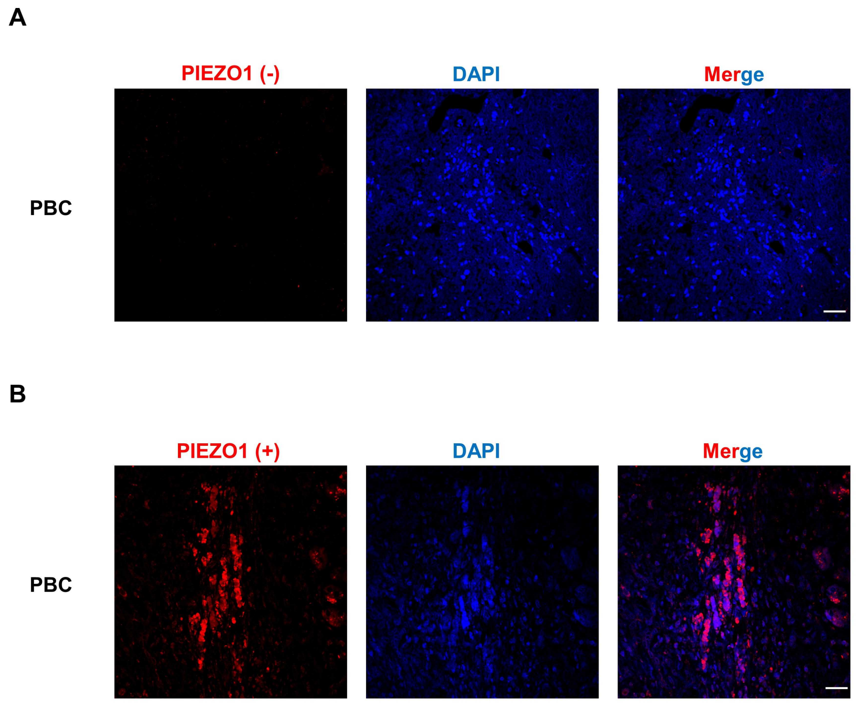

Fig S1. The specificity of the Piezo1 antibody was measured by immunofluorescence staining.

(A) Representative images of immunofluorescence staining without incubating with Piezo1 in human liver sample. **(B)** Representative images of immunofluorescence staining with Piezo1 (red) in human liver sample. Scale bar, 50 μm.

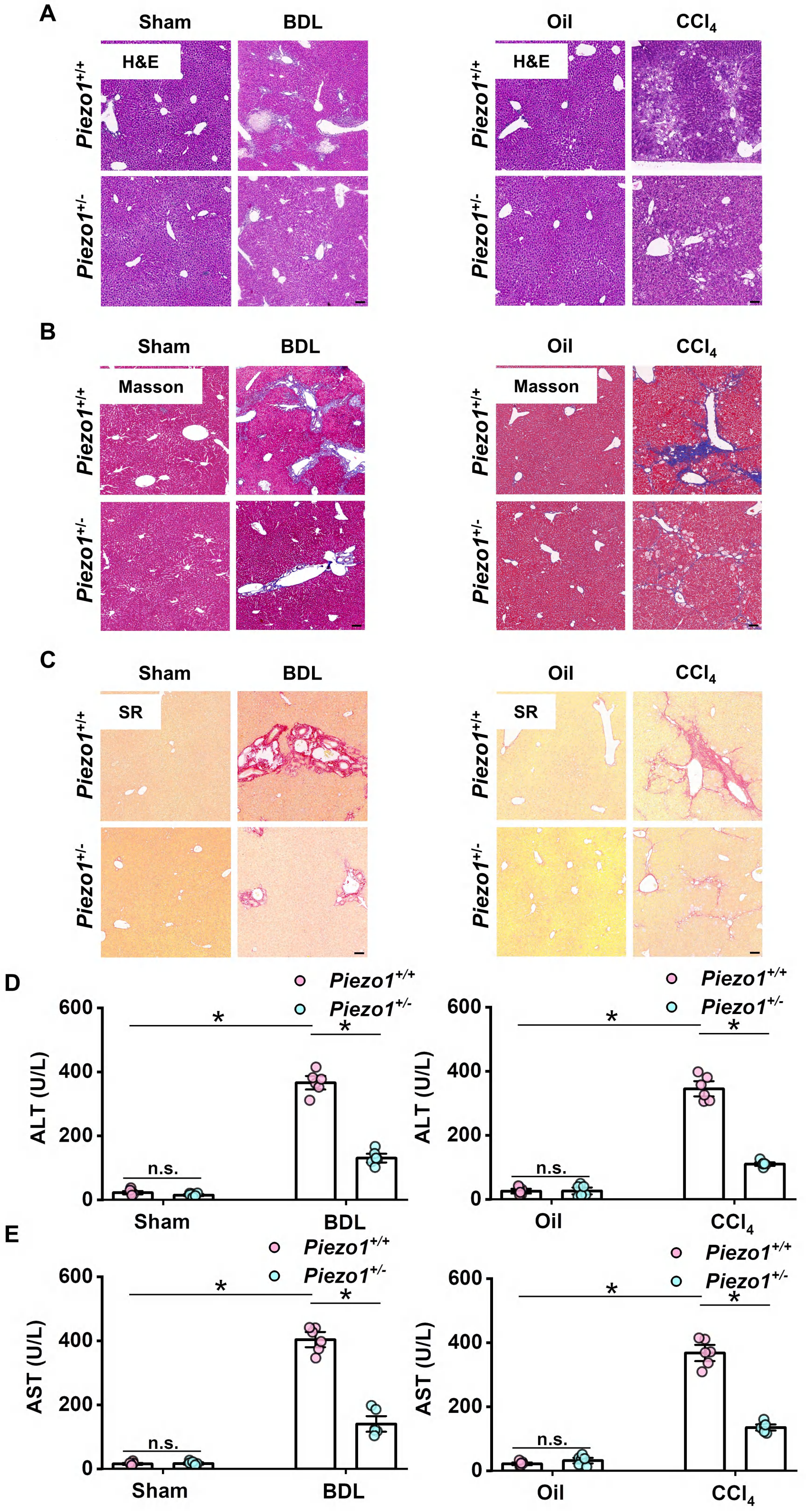

Fig S2. Liver fibrosis was attenuated in BDL-operated and CCl4-injected ***Piezo1***$^{+/-}$ **mice.** Representative images of **(A)** H&E, **(B)** Masson's and **(C)** Sirius red staining in the liver sections of *Piezo1*$^{+/+}$ and *Piezo1*$^{+/-}$ mice. Scale bar, 100 μm. **(D&E),** Serum concentrations of ALT and AST were measured in *Piezo1*$^{+/+}$ and *Piezo1*$^{+/-}$ mice. Data are presented as mean ± S.

E. M.; n = 6 for each group; *$P < 0.05$.

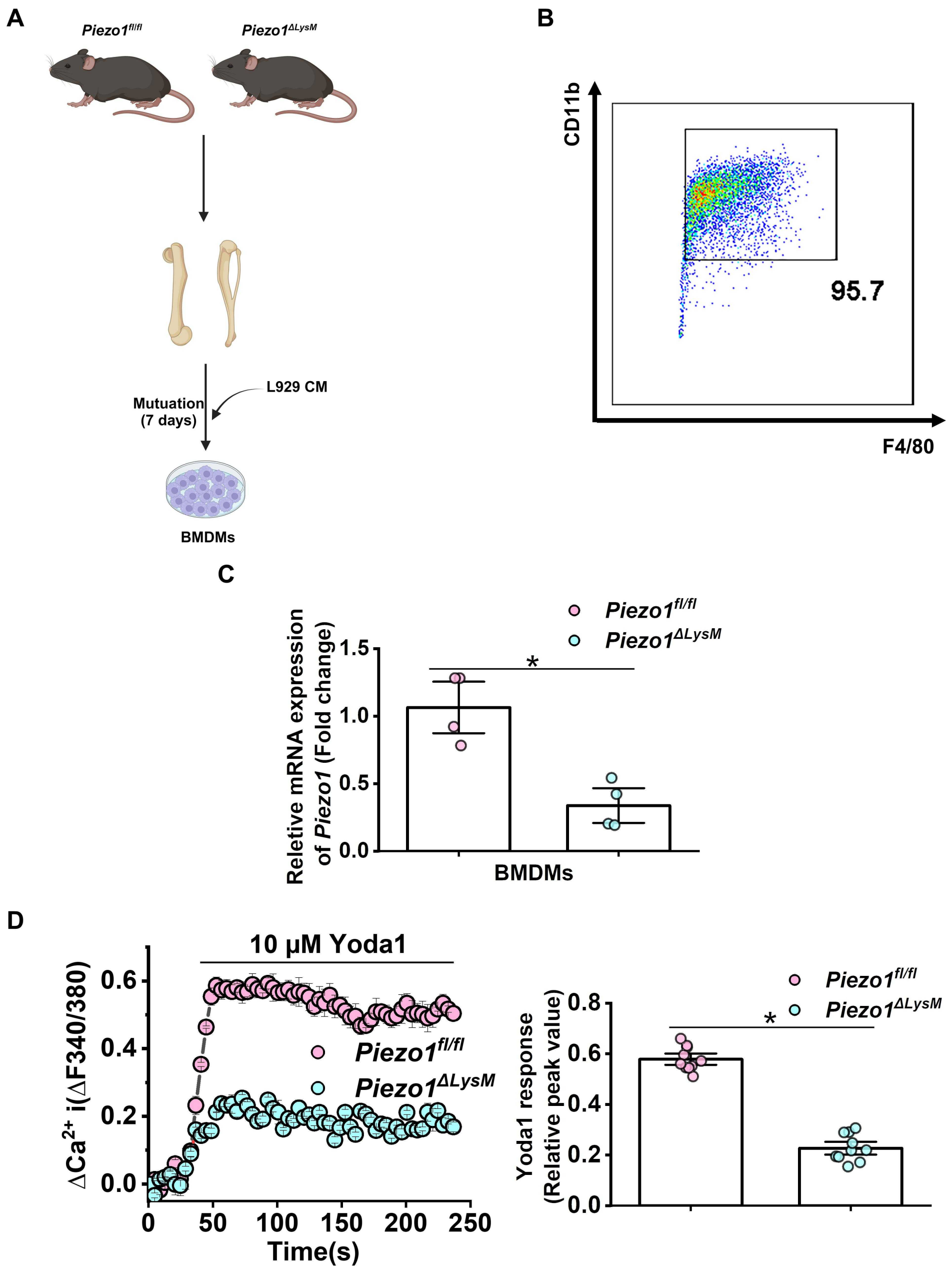

Fig S3. Validation of Piezo1 deletion in BMDMs.

(A) Schematic diagram of the isolation of BMDMs from *Piezo1*$^{fl/fl}$ and *Piezo1*$^{\triangle LysM}$ mice. **(B)** Purify of *Piezo1*$^{fl/fl}$ and *Piezo1*$^{\triangle LysM}$ BMDMs by FCM. **(C)** Relative mRNA expression of *Piezo1* in *Piezo1*$^{fl/fl}$ and *Piezo1*$^{\triangle LysM}$ BMDMs were measured. n = 4 for each group. **(D)** Experiment of Ca^{2+} influx and peak value in *Piezo1*$^{fl/fl}$ and *Piezo1*$^{\triangle LysM}$ BMDMs treated with 10 μM Yoda1 were measured (left: traces from the mean values of 8 wells for each group, right: n = 10 for the mean values of calcium measurements). Data are presented as mean ± S. E. M.; $^*P < 0.05$.

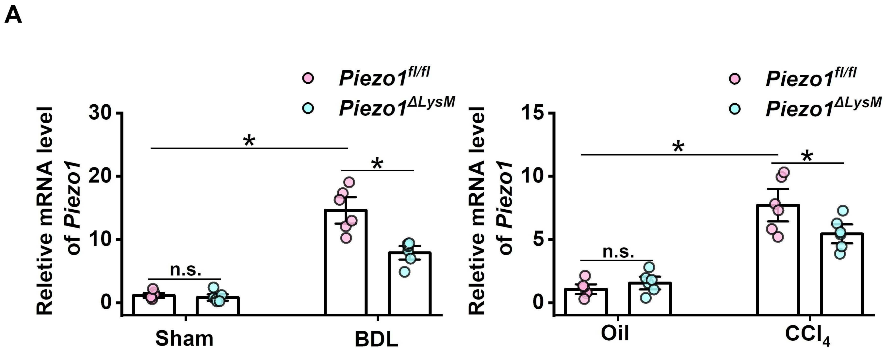

Fig S4. Piezo1 expression is increased in fibrotic livers.

(A) Relative mRNA expression of *Piezo1* in liver tissues of *Piezo1*$^{fl/fl}$ and *Piezo1*$^{\triangle LysM}$ mice were measured. n = 6 for each group. Data are presented as mean ± S. E. M.; *$P < 0.05$.

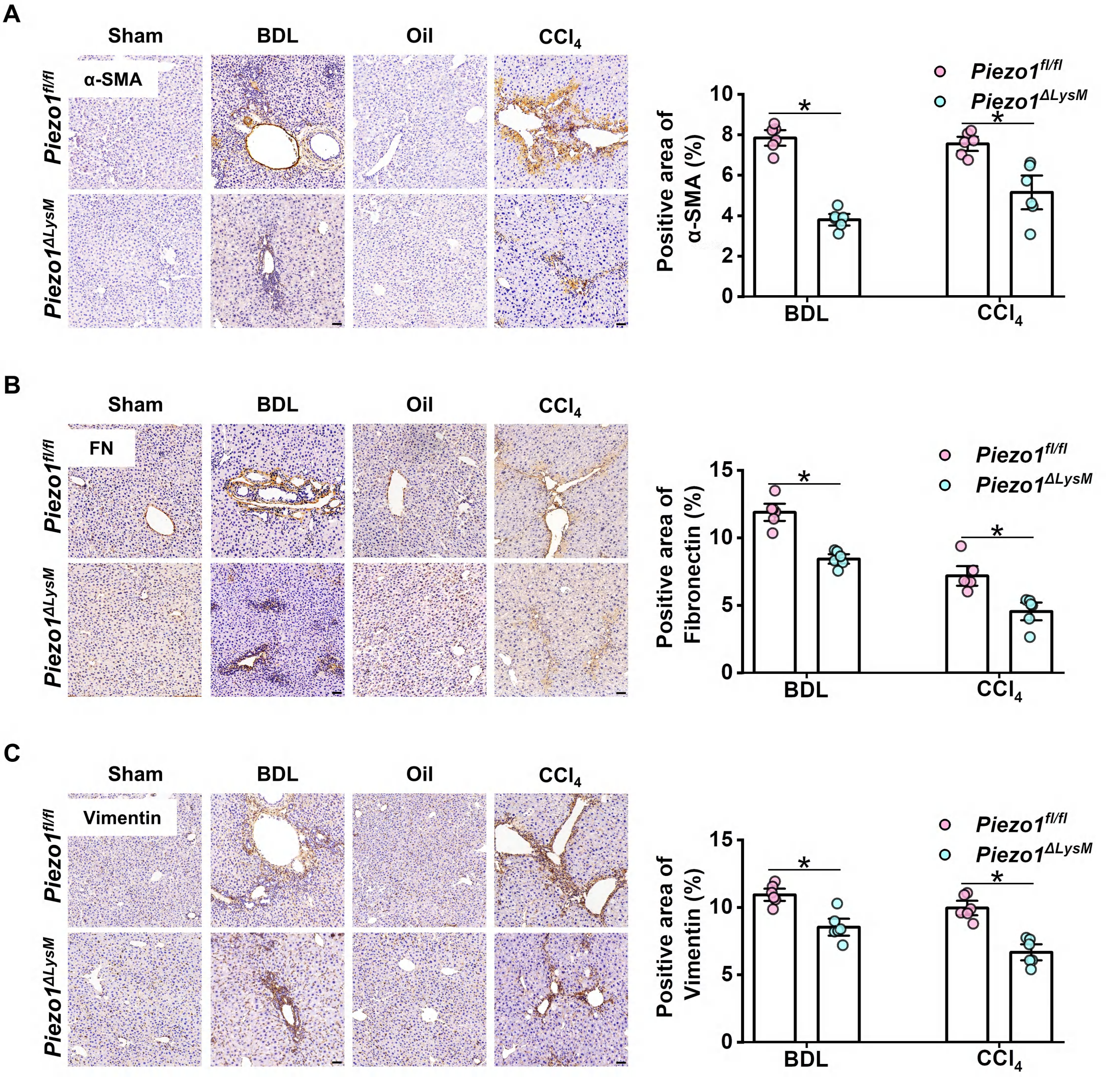

Fig S5. Myeloid-specific *Piezo1* deletion attenuates liver fibrosis.

Immunochemistry staining of **(A)** α-SMA, **(B)** FN (fibronectin) and **(C)** vimentin in the liver sections of $Piezo1^{fl/fl}$ and $Piezo1^{\triangle LysM}$ mice. Scale bar, 100 μm. Data are presented as mean ± S. E. M.; n = 6 for each group; *$P < 0.05$.

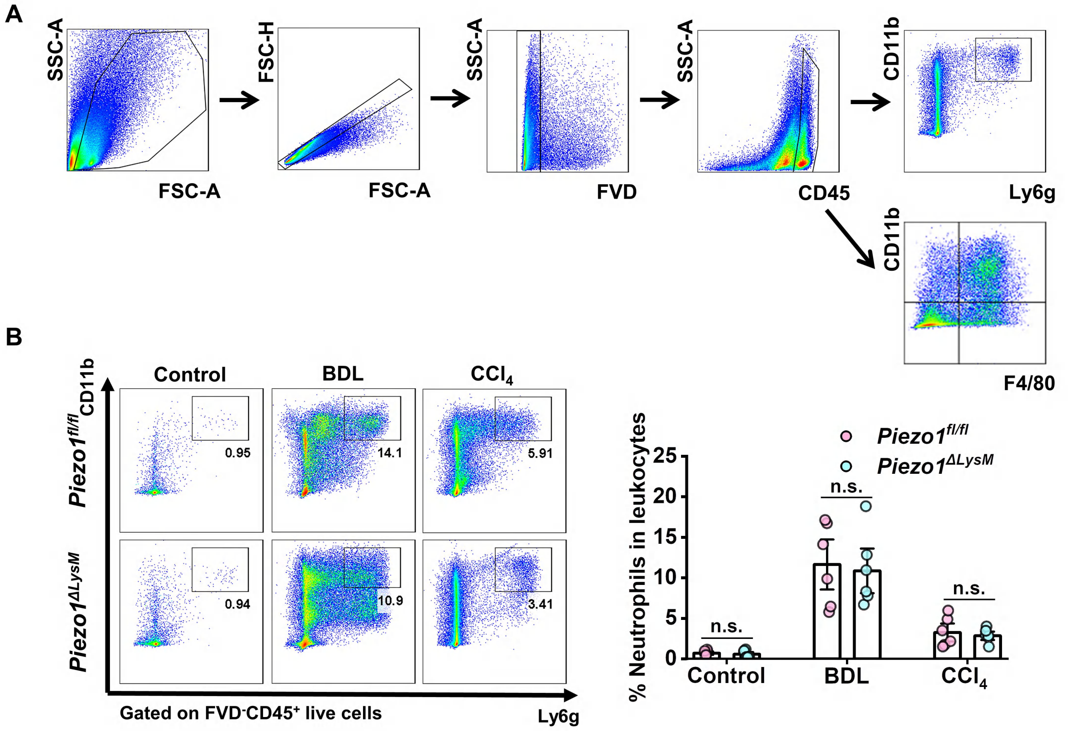

Fig S6. Myeloid-specific *Piezo1* doesn't influence neutrophils infiltration in murine fibrotic livers.

(A) FCM gating strategy showed liver myeloid cells. FVD$^-$ cells were gated as indicated for live cells. **(B)** Representative dot plot images showed the percentage of hepatic neutrophils (FVD$^-$CD45$^+$CD11b$^+$Ly6g$^+$) detected by FCM in *Piezo1*$^{fl/fl}$ and *Piezo1*$^{\triangle LysM}$ mice. Data are presented as mean ± S. E. M.; n = 6 for each group; **P < 0.05*.

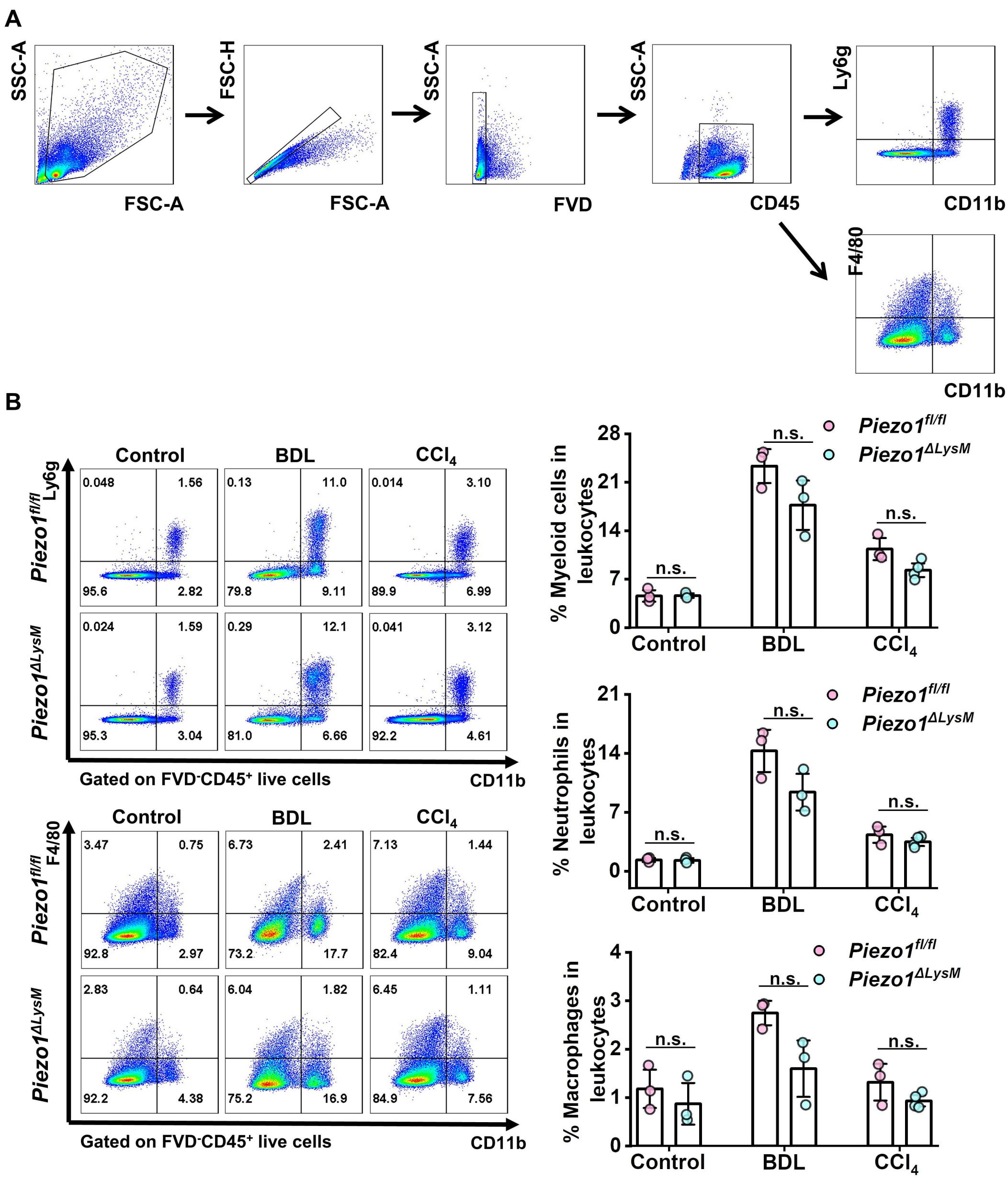

Fig S7. Myeloid-specific *Piezo1* doesn't affect the number of splenic myeloid cells.

(A) FCM gating strategy showed splenic myeloid cells. FVD$^-$ cells were gated as indicated for live cells. **(B)** Splenic myeloid cells (FVD$^-$CD45$^+$CD11b$^+$), neutrophils (FVD$^-$CD45$^+$CD11b$^+$Ly6g$^+$) and macrophages (FVD$^-$CD45$^+$CD11b$^+$F4/80$^+$) were examined. Data are presented as mean ± S. E. M.; n = 3-4 for each group; **P < 0.05*.

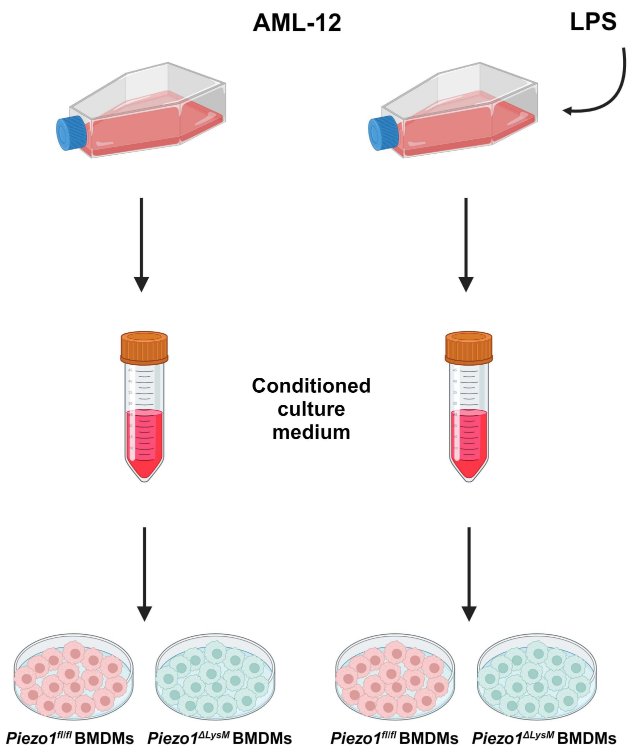

Fig S8. Schematic diagram of BMDMs treated with conditioned culture medium collected from AML-12.

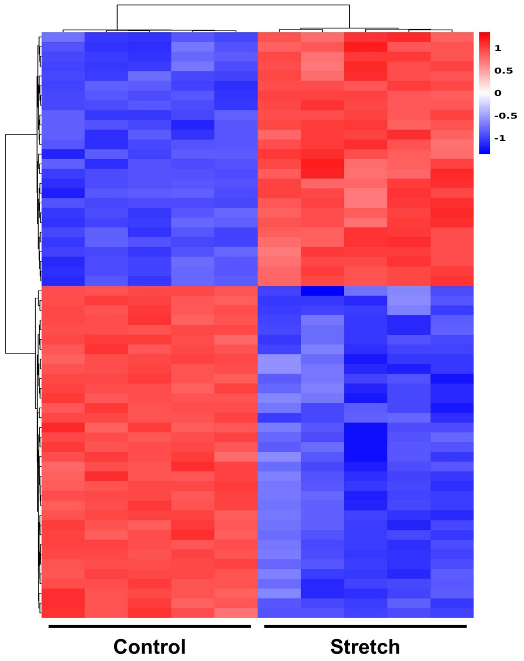

Fig S9. Heatmap exhibited that differentially expressed genes in stretch and control *Piezo1*$^{fl/fl}$ BMDMs.

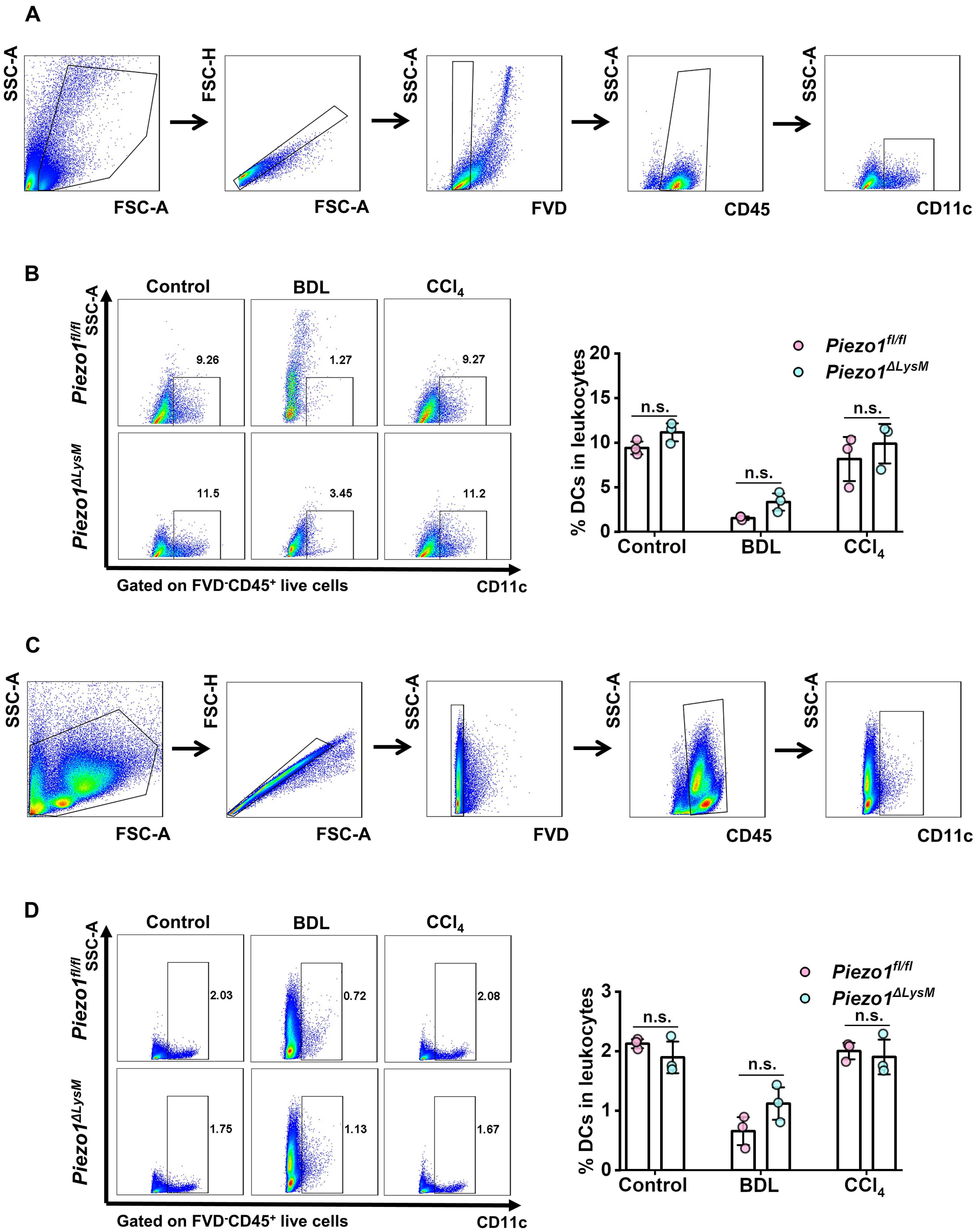

Fig S10. Myeloid-specific *Piezo1* doesn't affect hepatic and splenic dendritic cells infiltration in mice.

(A) FCM gating strategy showed liver dendritic cells. FVD$^-$ cells were gated as indicated for live cells. **(B)** Representative dot plot images showed the percentage of hepatic dendritic cells (FVD$^-$CD45$^+$CD11c$^+$) detected by FCM in *Piezo1*$^{fl/fl}$ and *Piezo1*$^{\Delta LysM}$ mice. **(C)** FCM gating strategy showed splenic dendritic cells. FVD$^-$ cells were gated as indicated for live cells. **(D)** Representative dot plot images showed the percentage of splenic dendritic cells (FVD$^-$

$CD45^+CD11c^+$) detected by FCM in *Piezo1*$^{fl/fl}$ and *Piezo1*$^{\triangle LysM}$ mice. Data are presented as mean ± S. E. M.; n = 3 for each group; *$P < 0.05$.

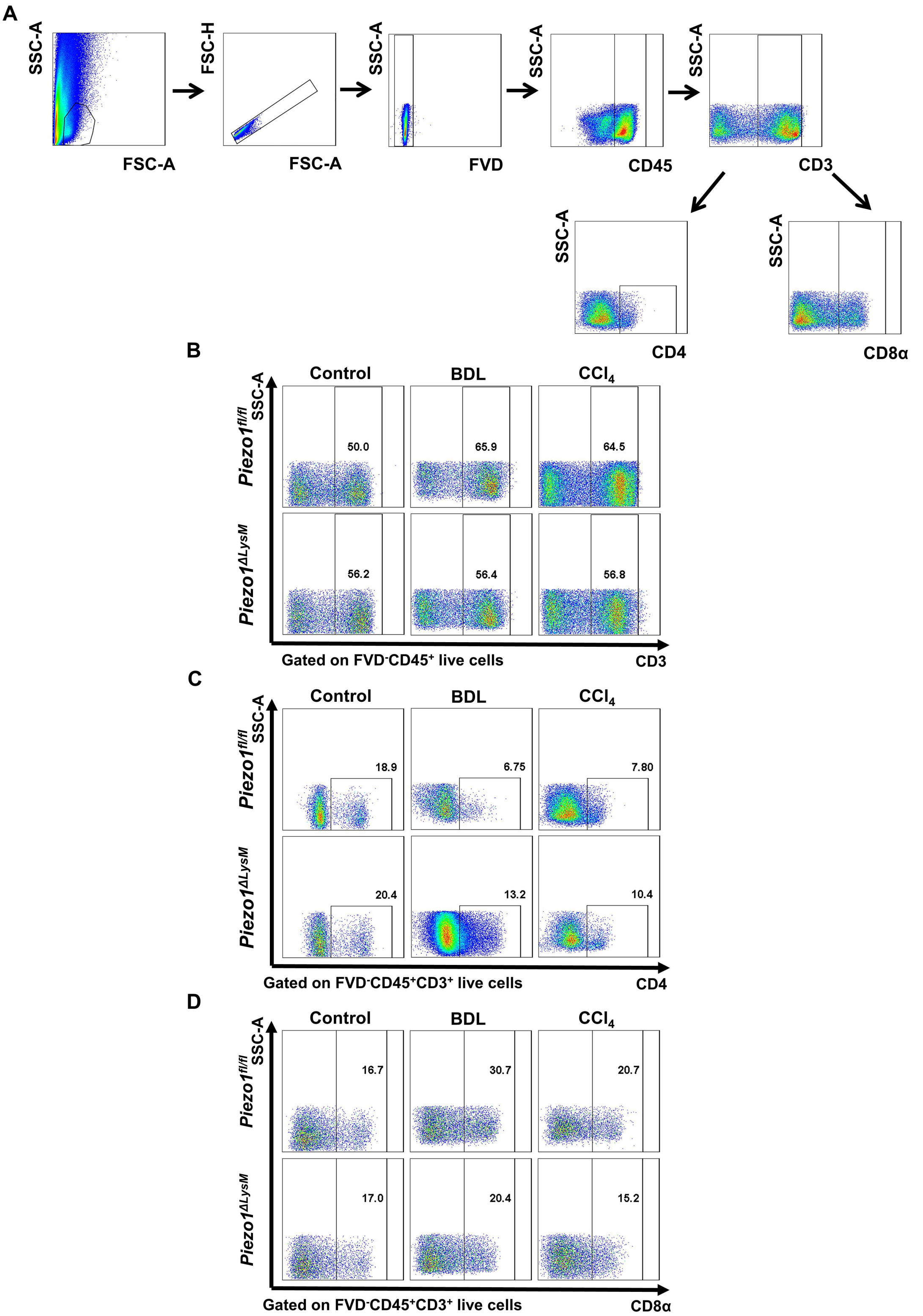

Fig S11. Liver T cells were shown by FCM gating strategy.

(A) FVD$^-$ cells were gated as indicated for live cells. The gating strategy of hepatic CD3$^+$T cells (FVD$^-$CD45$^+$CD3$^+$), CD4$^+$T cells (FVD$^-$CD45$^+$CD3$^+$CD4$^+$) and CD8$^+$T cells (FVD$^-$CD45$^+$CD3$^+$CD8$^+$) were shown. Representative dot plot images showed the percentage of

hepatic **(B)** $CD3^+$T cells, **(C)** $CD4^+$T cells and **(D)** $CD8^+$T cells detected by FCM in *Piezo1*$^{fl/fl}$ and *Piezo1*$^{\triangle LysM}$ mice.

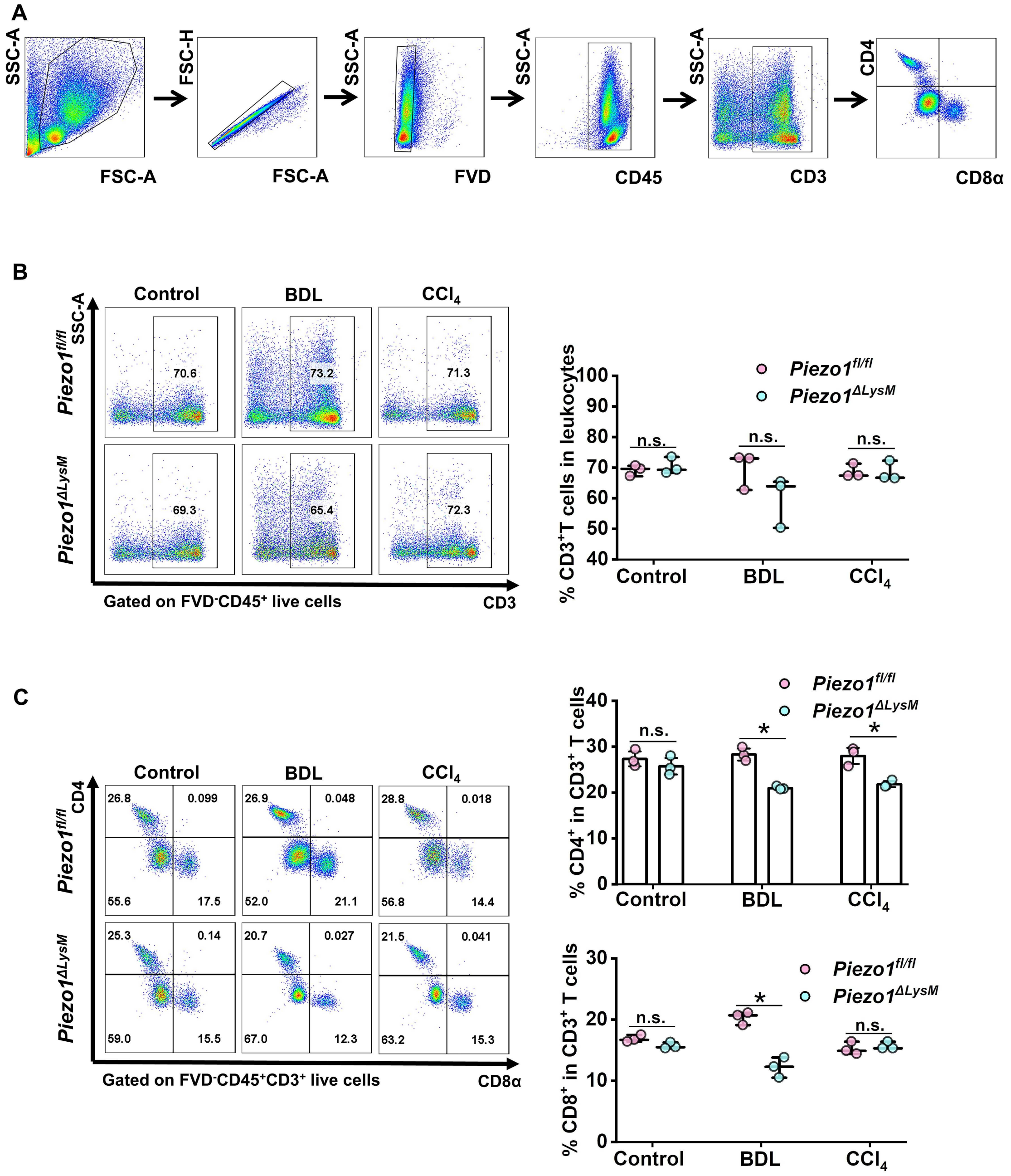

Fig S12. Splenic T cells were shown by FCM gating strategy.

(A) FVD$^-$ cells were gated as indicated for live cells. The gating strategy of splenic CD3$^+$T cells (FVD$^-$CD45$^+$CD3$^+$), CD4$^+$T cells (FVD$^-$CD45$^+$CD3$^+$CD4$^+$) and CD8$^+$T cells (FVD$^-$CD45$^+$CD3$^+$CD8$^+$) were shown. Representative dot plot images showed the percentage of splenic **(B)** CD3$^+$T cells, **(C)** CD4$^+$T cells and CD8$^+$T cells detected by FCM in *Piezo1$^{fl/fl}$* and *Piezo1$^{\triangle LysM}$* mice. Data are presented as mean ± S. E. M.; n = 3 for each group; **P < 0.05*.

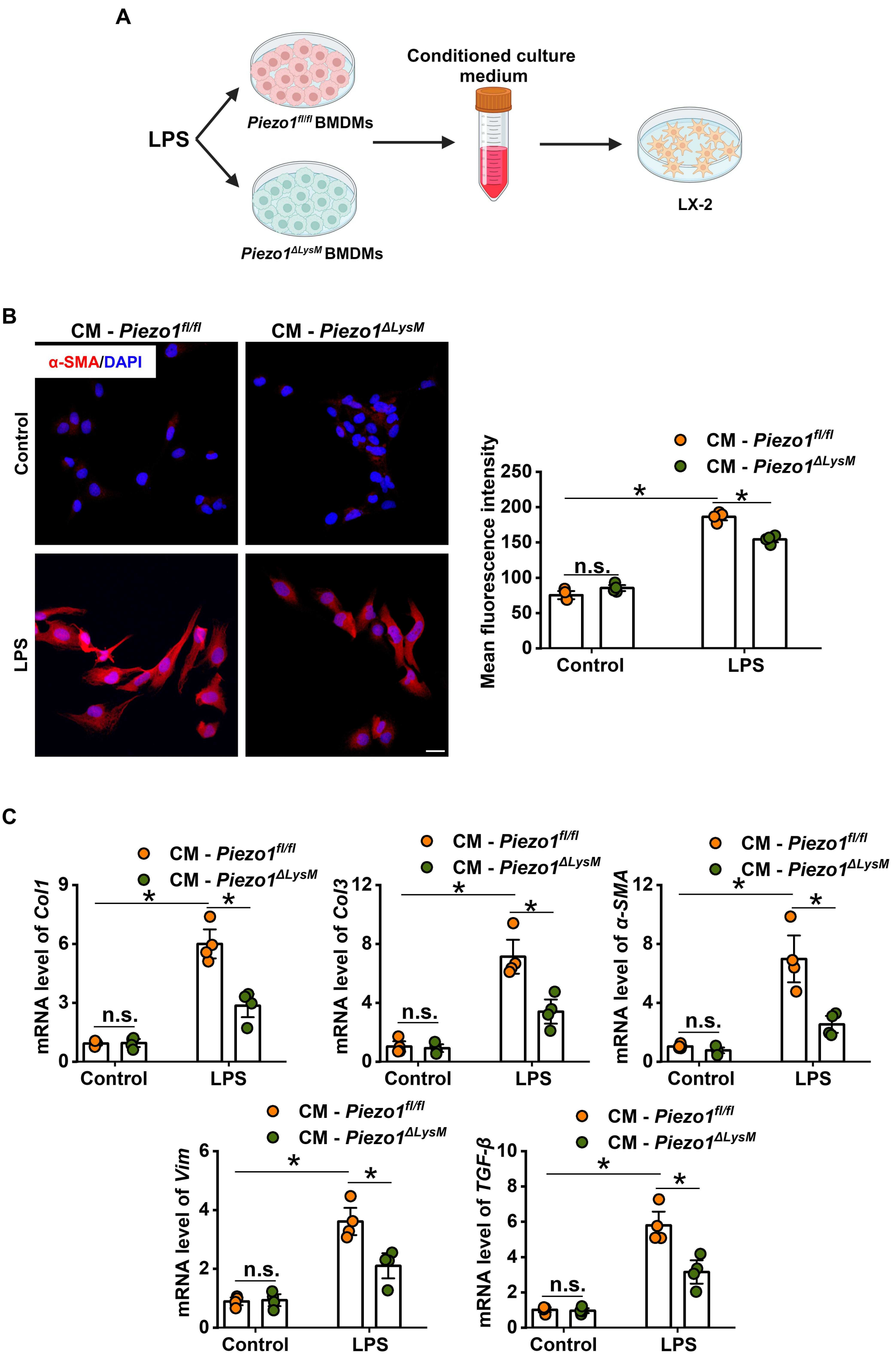

Fig S13. Myeloid-specific *Piezo1* regulates activation of human hepatic stellate cells (LX-2) through impairing macrophage inflammatory response.

(A) Schematic diagram of LX-2 cells treated with conditioned culture medium collected from *Piezo1*$^{fl/fl}$ and *Piezo1*$^{\triangle LysM}$ BMDMs. **(B)** Representative immunofluorescence images for α-

SMA (red) of LX-2 cells treated with conditioned culture medium from *Piezo1*$^{fl/fl}$ and *Piezo1*$^{\triangle LysM}$ BMDMs. Scale bar, 25 µm. **(C)** Relative mRNA expression of *Col1*, *Col3*, *α-SMA*, *Vim* and *TGF-β* in LX-2 cells were quantified. Data are presented as mean ± S. E. M.; n = 4 for each group; **P < 0.05*.

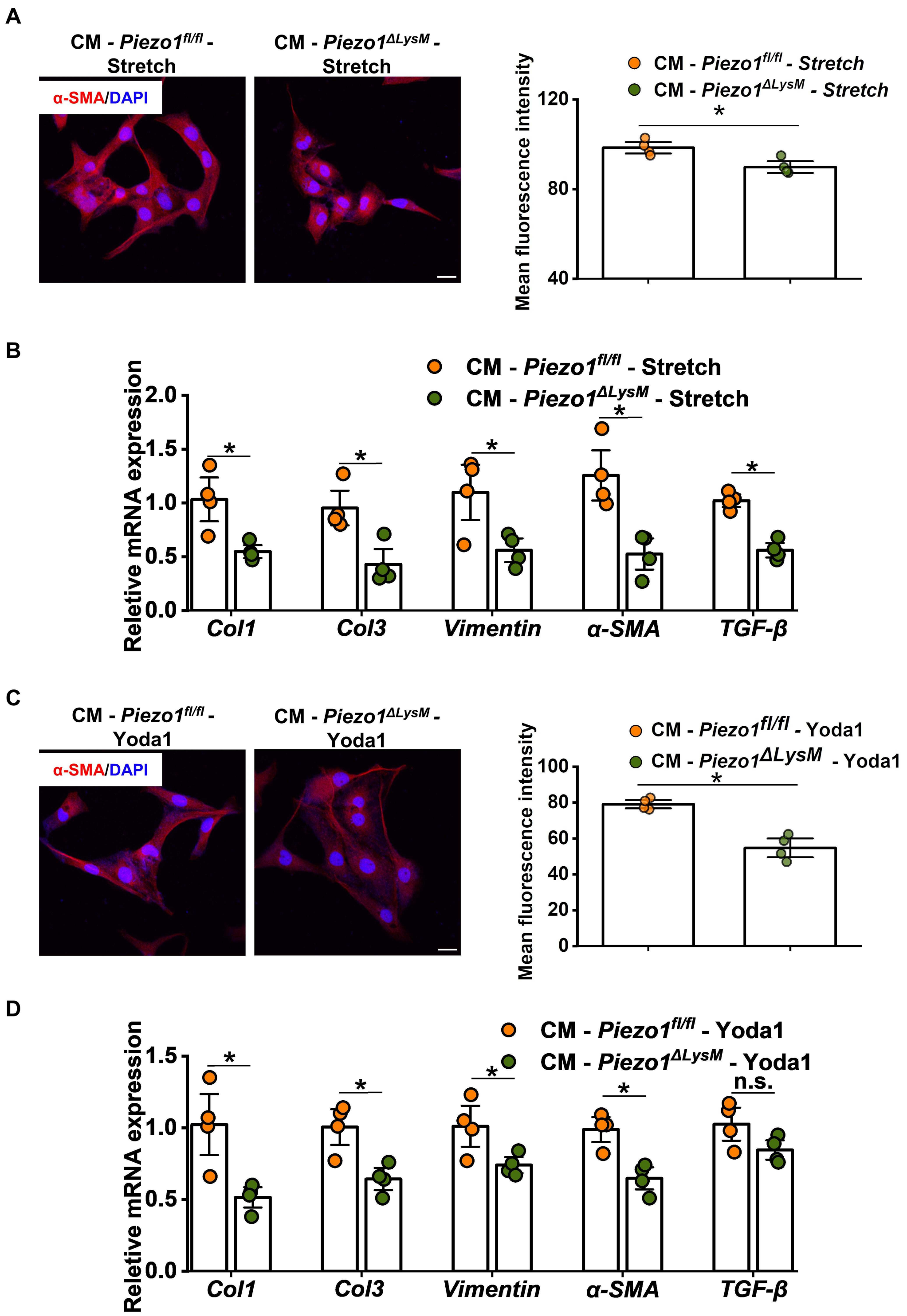

Fig S14. Myeloid-specific *Piezo1* deletion inhibits activation of LX-2.

(A) Representative immunofluorescence images for α-SMA (red) of LX-2 cells treated with conditioned culture medium collected from *Piezo1*$^{fl/fl}$ and *Piezo1*$^{\triangle LysM}$ BMDMs. Scale bar, 25 μm. **(B)** Relative mRNA expressions of *Col1, Col3, α-SMA, Vim* and *TGF-β* in LX-2 cells were quantified. **(C)** Representative immunofluorescence images for α-SMA (red) of LX-2 cells treated with conditioned culture medium collected from *Piezo1*$^{fl/fl}$ and *Piezo1*$^{\triangle LysM}$

BMDMs. Scale bar, 25 μm. **(D)** Relative mRNA expressions of *Col1, Col3, α-SMA, Vim* and *TGF-β* in LX-2 cells were quantified. Data are presented as mean ± S. E. M.; n = 4 for each group; *$P < 0.05$.

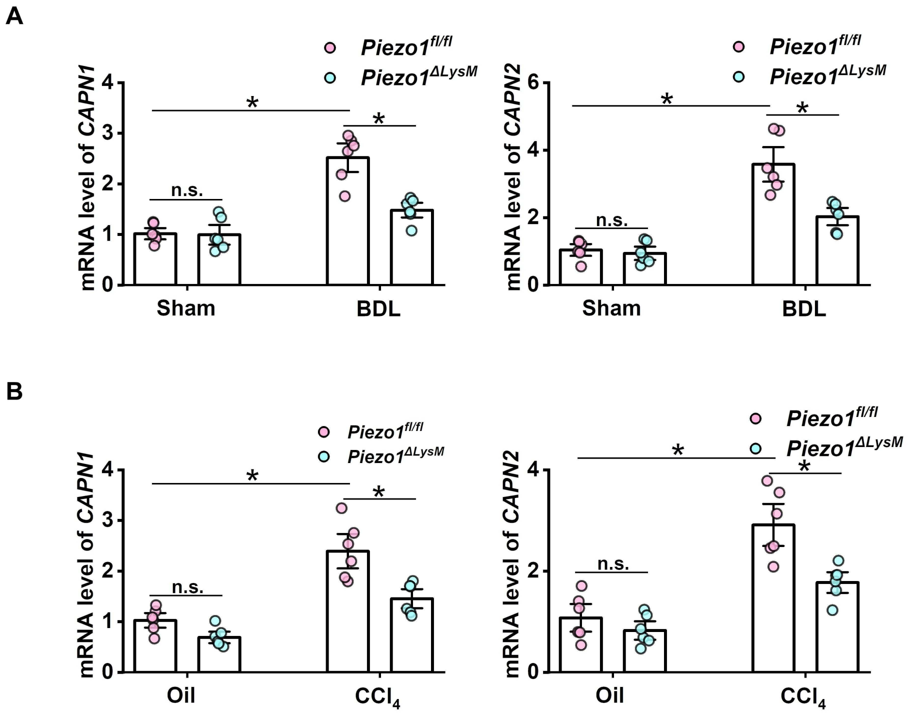

Fig S15. Myeloid-specific *Piezo1* regulates calpain activity in murine fibrotic livers.

(A) Relative mRNA expressions of *CAPN1* and *CAPN2* in liver tissues of sham-operated or BDL-operated mice were quantified. **(B)** Relative mRNA levels of *CAPN1* and *CAPN2* in liver tissues of Oil or CCl_4-injected mice were quantified. Data are presented as mean ± S. E. M.; n = 6 for each group; *$P < 0.05$.

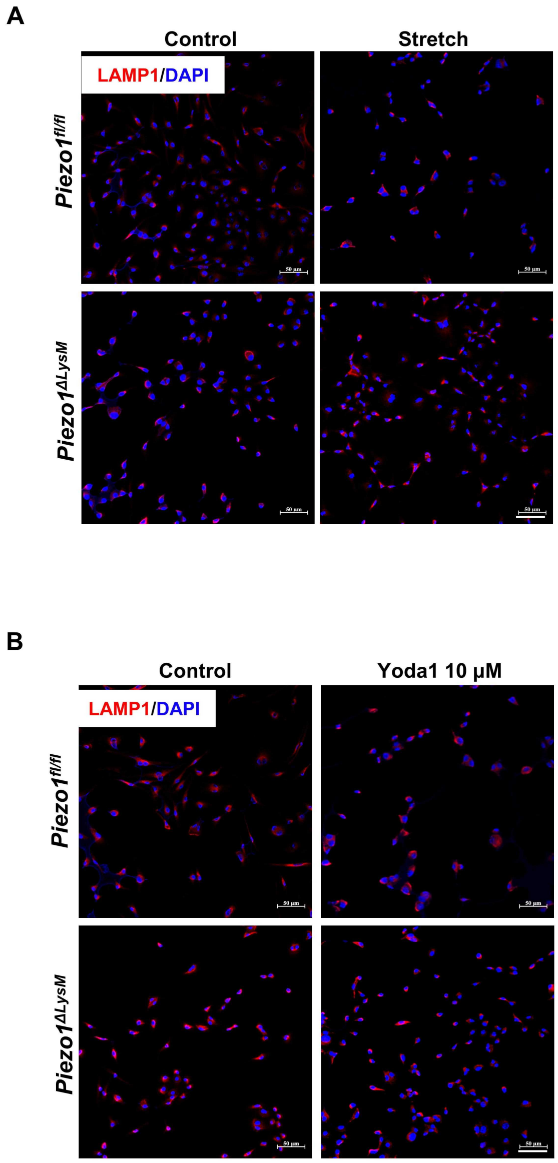

Figure S16. Activation of Piezo1 controls degradation of LAMP1.

(A) Immunofluorescence staining with LAMP1 (red) in *Piezo1*$^{fl/fl}$ or *Piezo1*$^{\triangle LysM}$ BMDMs treated with mechanical stretch compared with controls were shown. **(B)** Immunofluorescence staining with LAMP1 (red) in *Piezo1*$^{fl/fl}$ or *Piezo1*$^{\triangle LysM}$ BMDMs treated with Yoda1 compared with controls were shown. Scale bar, 50 µm. n = 3 for each group.
